# Supplementary material for: Genetic isolation and metabolic complexity of an Antarctic subglacial microbiome
Source: Nat Commun. 2025 Aug 18;16:7501. doi: 10.1038/s41467-025-62753-3 (PMC12361377; doi:10.1038/s41467-025-62753-3)
Supplement: Supplementary file 1 — Supplementary Information [file 41467_2025_62753_MOESM1_ESM.pdf]

# Supplementary information

## Genetic isolation and metabolic complexity of an Antarctic subglacial microbiome

Kyung Mo Kim, Kyuin Hwang, Hanbyul Lee, Ahnna Cho, Christina L. Davis, Brent C. Christner, John C. Priscu and Ok-Sun Kim

## Supplementary Note

**Note 1. Single-cell genomes recovered from SLM**

In late December 2018, an environmentally clean hot water drill system was used to melt a ~0.4 m diameter borehole through 1,087 m of ice to study SLM[1]. Particles in the water were concentrated and fractionated into four subsamples based on diameter (>3 µm, 3 to 0.8 µm, 0.8 to 0.2 µm, and <0.2 µm) using an *in-situ* filtering system. Five samples were collected from the top 10 cm of a sediment core (i.e. Unit I[2]) that was split every 2 cm. In addition, bulk surface sediment collected by a sediment catcher attached to the base of the *in-situ* water filtration system were also analyzed. The cell concentration in the lake water (2.4 x $10^4$ cells $mL^{-1}$) and surficial sediments (2.6 x $10^6$ cells $g^{-1}$) were determined as previously described[2]. Owing to low concentrations of extractable DNA from the samples (0.70 ± 0.90 ng per extraction; ±SD), sequence libraries generated by NovaSeq 6000 sequencing (Illumina) assembled into a large number of short-length contigs with low sequence coverage[3]. To overcome this issue, we used a single-cell genomic approach.

From the four water and six sediment samples, a total of 3,170 droplets were sorted using flow cytometry[4]. DNA was successfully amplified from 2,431 isolated cells of the sorted droplets. After evaluating quality based on crossing point (Cp) values, 2,000 of the cells were selected for genome sequencing, which yielded an average of 8.94 million paired reads per cell (Supplementary Data 1). Filtering to remove reads of low-quality, of low-complexity, and identified as human DNA contamination discarded an average of 6% of the reads per cell. After discarding sequences affected by genome amplification bias[5], 4.2% of the remaining reads (367,552 read pairs per cell on average) were retained. Of the 2,000 cells selected for genome sequencing, 605 failed to provide sufficient data for successful assembly or taxonomic

classification of the sequences. Following removal of 21 SAGs that exhibit >5% CheckM contamination rate[6], a total of 1,374 SAGs (618 from the water column and 756 from the sediment samples) were available for downstream analyses. After removing the foreign contaminated sequences using NCBI's Foreign Contamination Screen tool suite[7], the SAGs are composed of 79 ± 50 (average ± standard deviation) contigs with a read coverage of 65.2 ± 6.5 ×, length of 997,887 ± 608,779 bp, GC content of 57.1 ± 9.1%, CheckM completeness of 38.7 ± 18.8%, and CheckM contamination of 0.4% ± 0.7%.

**Note 2. Factors leading to different community structures between SAG and ASV datasets**

When 16S rRNA gene sequences of SAGs were compared with amplicon data from SLM, SLW and WGZ samples[2], taxa vary in composition and in abundance between the method (Supplementary Fig. 2b-c). This difference appeared prominently in the genera *Thiobacllius* and *Sideroxydans*. The genus *Thiobacillus* (6.43% of total ASVs) were among the most abundant taxa in sediments of SLM, while none of the SAGs possessed 16S rRNA sequences similar to *Thiobacillus* ASVs nor even belonged to the family Thiobacillaceae in the GTDB taxonomy. This difference would be attributed to inherent technical bias of both 16S rRNA gene amplification and single-cell genomics (see details in Results). In addition, the *Thiobacillus* ASVs were absent or rare in 13 of 58 sediment samples of SLM, indicating that the taxon composition can vary largely across samples. Therefore, the differential community structure between SAGs and ASVs would also partly results from sampling bias.

The *Sideroxydans* taxa (2.18% of total ASVs in SLM) that have been considered dominant in Antarctic subglacial environments[2] were not present in the GTDB taxonomy of SAG (Supplementary Data 1). We hypothesized that this discrepancy between SAG and ASV datasets results from differences in taxonomic classification systems since the ASV sequences were annotated using the SILVA v138 database[2]. We reconstructed a phylogenetic tree to determine whether 16S rRNA sequences from the SAGs cluster with ASVs and the SILVA sequences affiliated with *Sideroxydans*. Using SINA v1.7.2[8], we aligned 16S rRNA sequences from four datasets: derived from the following datasets: 1) SAGs classified in GTDB as the family Gallionellaceae (64 16S rRNA sequences), 2) GTDB representative genomes within Gallionellaceae (68 sequences), 3) ASVs within Gallionellaceae (143 sequences), and 4) SILVA sequences assigned to *Sideroxydans* (332 sequences). The poorly aligned sequences were

removed, and the V4 region was extracted for analysis. A resulting alignment of 253 sites was used to reconstruct a maximum-likelihood tree in RAxML v8.2.12[9], using TPM1uf+G4 substitution model[10] and 100 bootstrap replicates (Supplementary Fig. 5). We identified a clade containing six SAG-derived 16S rRNA sequences affiliated with the genus 39-52-13, which clustered with a SILVA sequence (EU030485) annotated as *Sideroxydans*, along with seven ASVs that showed the high sequence similarity (98.4% or more) to *Sideroxydans* SILVA sequences (Supplementary Fig. 5b). These results suggest that the 39-52-13 group in GTDB taxonomically corresponds to *Sideroxydans* and that this genus is present in both single-cell and ASV datasets.

**Note 3. Genetic heterogeneity among SAGs of the same species**

The KEGG orthologs (KOs) of closely related SAGs were examined to evaluate gene content within divergent microbial populations considered to belonging to the same species taxon. For this comparison, we first defined the null hypothesis as follow: any strains belonging to the same species should possess the same gene content when their complete genomes were compared. By surveying our single cell genomes, we found that the three genomes of the genus F1-60-MAGs149 is most suitable for this test. The genomes are similar each other with >98% ANI and that are >87% complete (SLM_LV1_BS-J08 of 87.1% CheckM completeness, SLM_MC1B_46-P03 of 90.4%, and SLM_MC1B_24-O19 of 90.9%; Supplementary Data 1). When the genome incompleteness was considered, the expected proportion of genes shared by the three genomes under the null hypothesis should be like a Venn diagram of Supplementary Fig. 28a. We assumed 1,550 KEGG orthologs as the average expected number of KO genes when the three incomplete genomes are considered complete. To assess whether the observed proportion deviated from the null expectation, we approximated the expected distribution using a Monte Carlo test with 10,000 iterations, in which, for each genome in every iteration, a number of KEGG orthologs equal to 1,550 multiplied by the genome completeness was randomly sampled. As a result, the observed proportion of KO genes shared by the genomes (52.3% in Supplementary Fig. 28b) was largely lower than the expected number (71.6%), rejecting the null hypothesis ($P$<0.01). Consequently, remarkable genetic heterogeneity exists among closely related genomes of the same species, which aligns well a general knowledge that a microbial species consists of a number of strains that vary in

functional traits[11]. Therefore, we here analyzed individual genomes at the strain level.

**Note 4. Metabolic potential of mineralization in SLM**

Metabolic pathways for nitrogen mineralization were represented in 113.3% of the lake_SAGs and 151.5% of the sediment_SAGs. In particular, urea hydrolysis (*ureABC* or *URE;* 48.9% lake_SAGs and 61.8% sediment_SAGs), urea amidolyase (*UCA-atzF;* 10.6% and 23.3%), nitrile deamination (*NIT;* 8.0% and 35.4%), nitroalkane deamination (*ncd2*, 64.0% and 95.0%), glutamate deamination (*gltS* or *gudB;* 7.1% and 4.0%) and cyanide deamination (*cynS;* 7.1% and 2.8%) were identified in the SAG dataset. The potential for sulfur mineralization was exhibited by 95.6% of the lake_SAGs and 141.3% of the sediment_SAGs. Specifically, cysteine/cysteate deaminase (*dcyD* or *cuyA;* 12.4% and 20.8%), taurine dioxygenase (*tauD;* 32.9% and 10.6%), sulfatase (*betC* or *aslA;* 48.9% and 77.9%), and alkanesulfonate monooxygenase (*ssuD;* 16.9% and 49.7%), were observed in the SAG dataset.

**Note 5. Inaccurate divergence time estimation resulting from differential generation-time**

Dating the evolutionary origins of these populations could be resolved by estimating the divergence time between the subglacial microorganisms and their closest non-subglacial neighbors. Relative to external biomes, microorganisms inhabiting low energy environments like Antarctic subglacial lakes are known to grow at a much slower pace[12]. The longer doubling time leads to slower DNA replication, which then produces the lower mutation rate[13]. Although it is technically feasible to use recent phylogenomic approaches to examine divergence times of taxonomic groups[14], existing uncertainties on the reproduction and mutation rates in these subglacial populations make accurate estimates challenging.

## Supplementary Figures

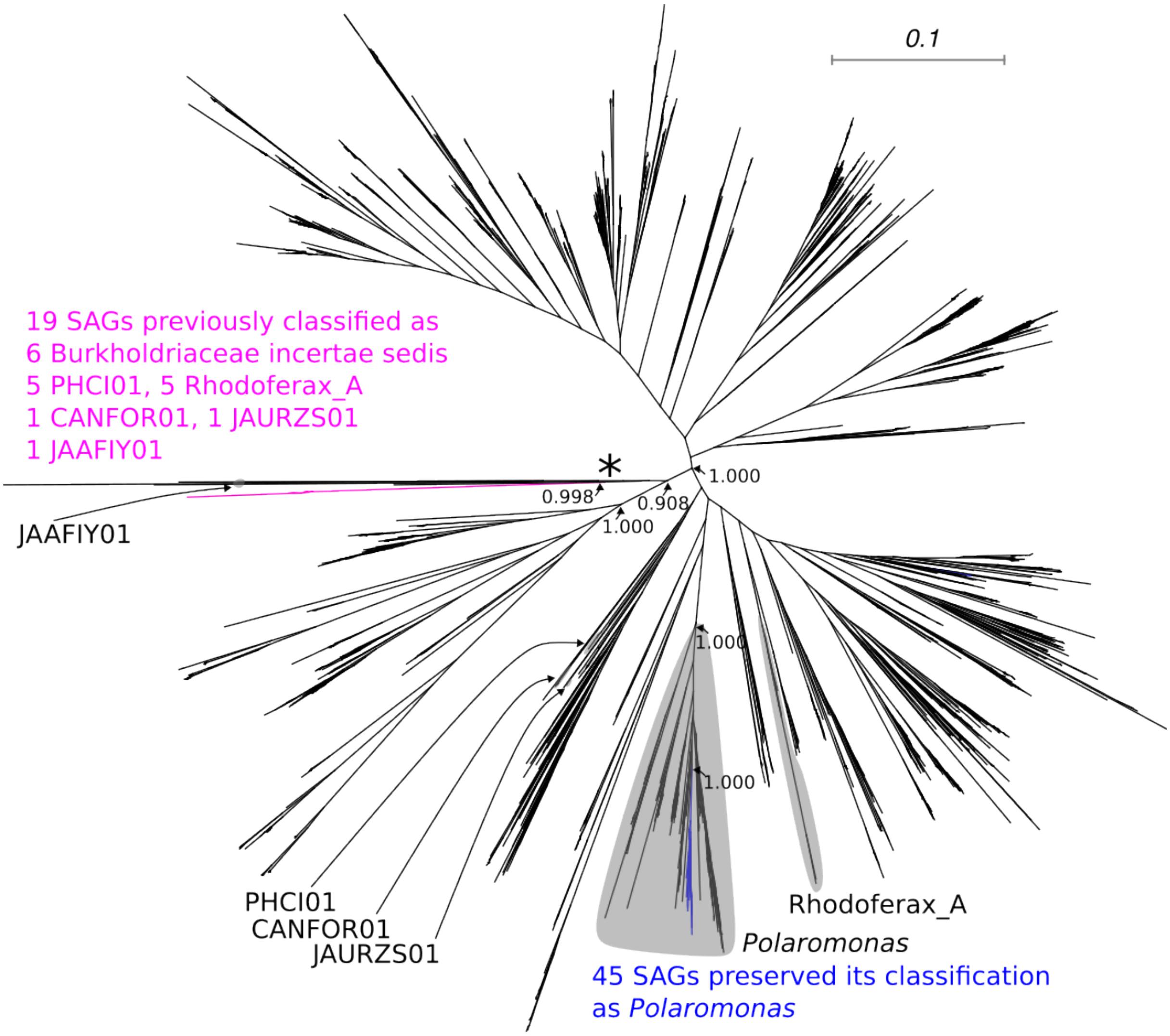


**Supplementary Fig. 1. An example of manual taxonomic correction.** The figure above is part of a maximum-likelihood tree reconstructed using the GTDB alignment of the family Burkholderiaceae that was cropped out at an asterisk. The 45 SLM's SAGs taxonomically affiliated with *Polaromonas* (branch in blue) form a monophyletic clade with other *Polaromonas* GTDB reference genomes. However, the 19 SAGs were cohesively clustered in this tree (magenta), but they were affiliated with different GTDB genera, each occupying distinct phylogenetic positions (e.g., PHCI01, CANFOR01). Based on the tree topology, these SAGs were taxonomically considered Burkholderiaceae incertae sedis in this study. The Shimodaira-Hasegawa support values for node confidence are presented next to the branches.

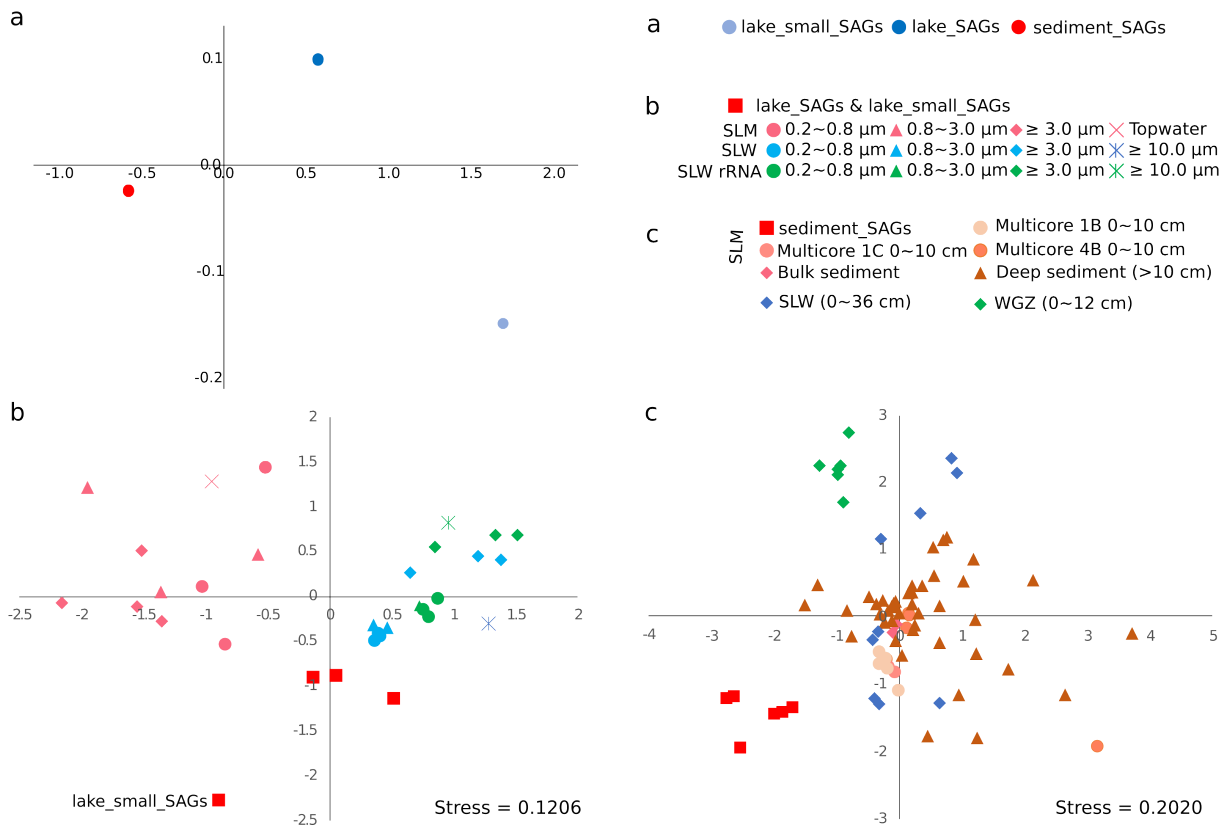


**Supplementary Fig. 2. Bray-Curtis dissimilarity-based non-metric multidimensional scaling between samples.** (**a**) NMDS of 10 subsamples of SLM, where three (>0.2 µm pore sizes, called lake_SAGs on figure) and one (<0.2 µm, lake_small_SAGs) from the water column and six from sediment (sediment_SAGs). The relatedness was based on the relative abundance of SAGs at the GTDB genus level. Note that the three water column samples of >0.2 um pore sizes and six sediment samples overlapped, respectively. (**b**) NMDS of lake water samples from this study (SAGs) and previous study (16S rDNA for SLM and SLW[2], and rRNA for SLW[15]). The relatedness between samples were scaled by relative abundance of OTUs. (**c**) NMDS of sediment samples from this study (SAGs) and previous study (16S rRNA for SLM, SLW, and WGZ)[2]. The detailed methods for drawing NMDS plots can be found in Methods. Source data are provided as a Source Data file.

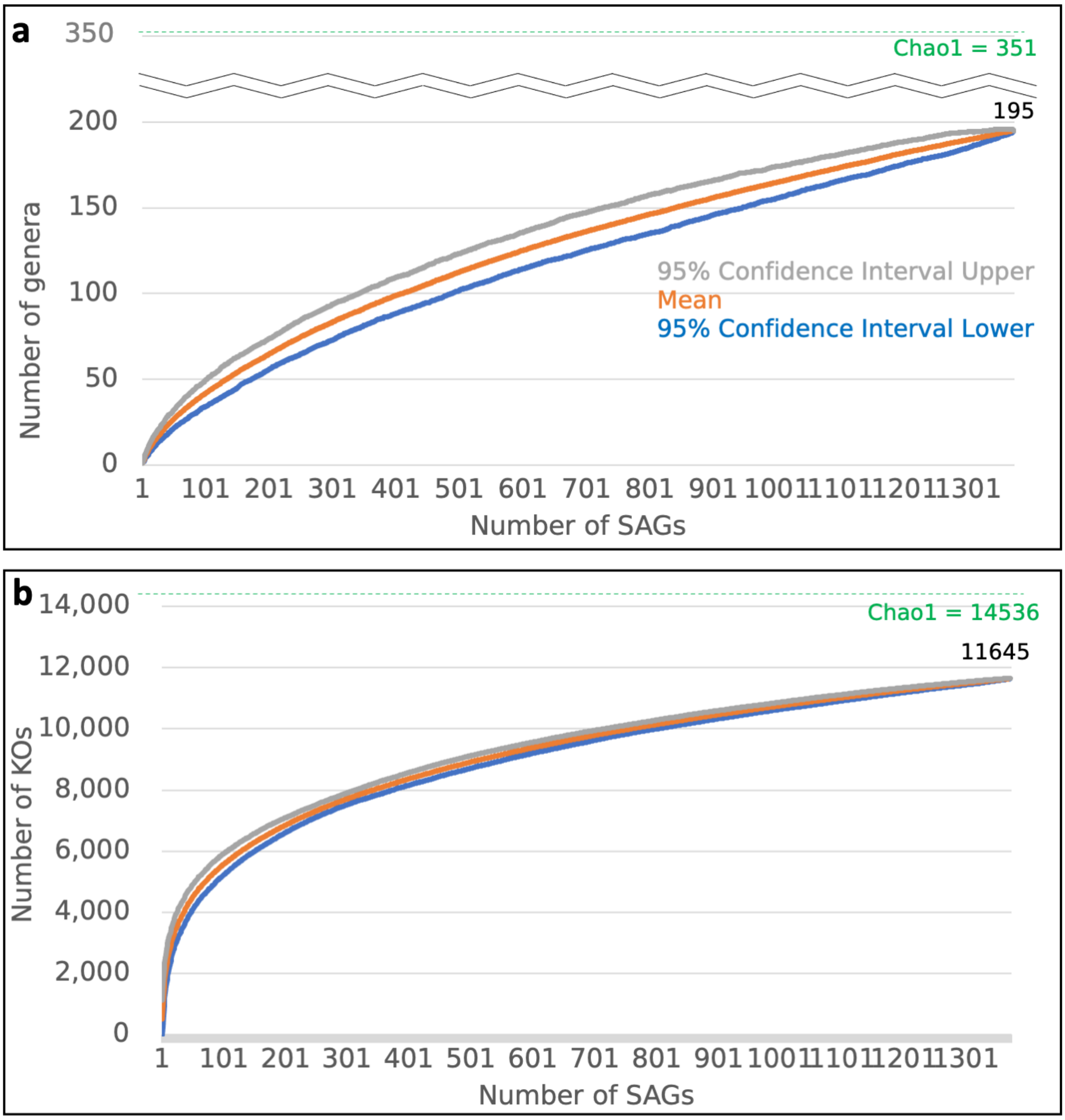


**Supplementary Fig. 3. The richness estimation for species diversity and functional diversity.** (**a**) the taxonomic richness at the GTDB genus level was evaluated using Chao1 (a horizontal green dotted line) and rarefaction with 95% confidence interval. (**b**) the functional richness at the KEGG orthology (KO) level evaluated using the same approach of (**a**). Source data are provided as a Source Data file.

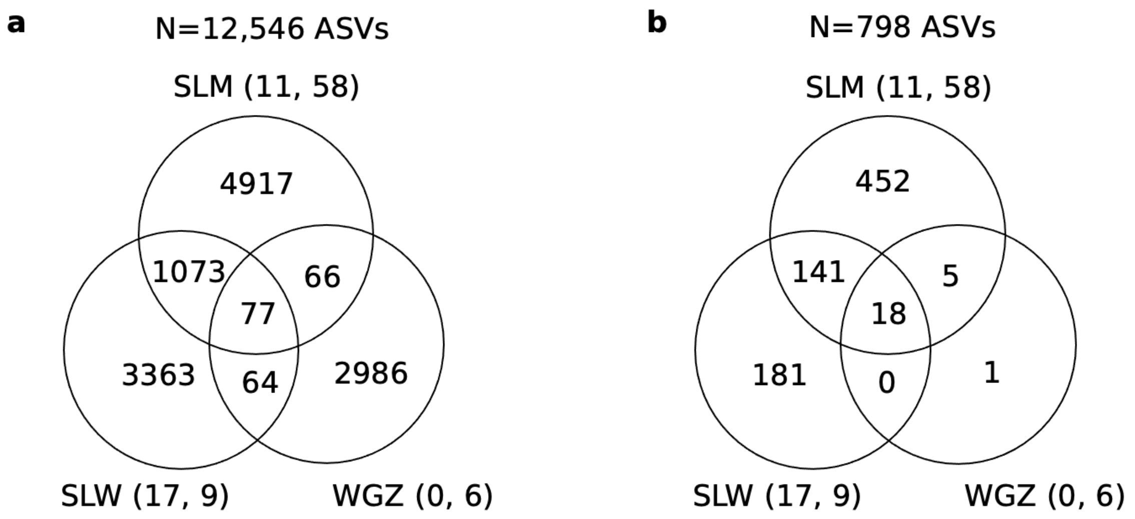


**Supplementary Fig. 4. Sharing ASVs between samples.** (**a**) previously sequenced ASVs shared by SLM, SLW, and WGZ[2]. The letter on the top of Venn diagram indicates the total number of ASVs. The numbers in parentheses indicate the numbers of samples from lake water and sediment, respectively. The 464 ASVs were only found in technical test samples that include the borehole water. (**b**) ASVs shared by SLM, SLW, and WGZ only for 803 ASVs that are nearly identical to 16S rRNA sequences extracted from SLM's SAGs. Source data are provided as a Source Data file.

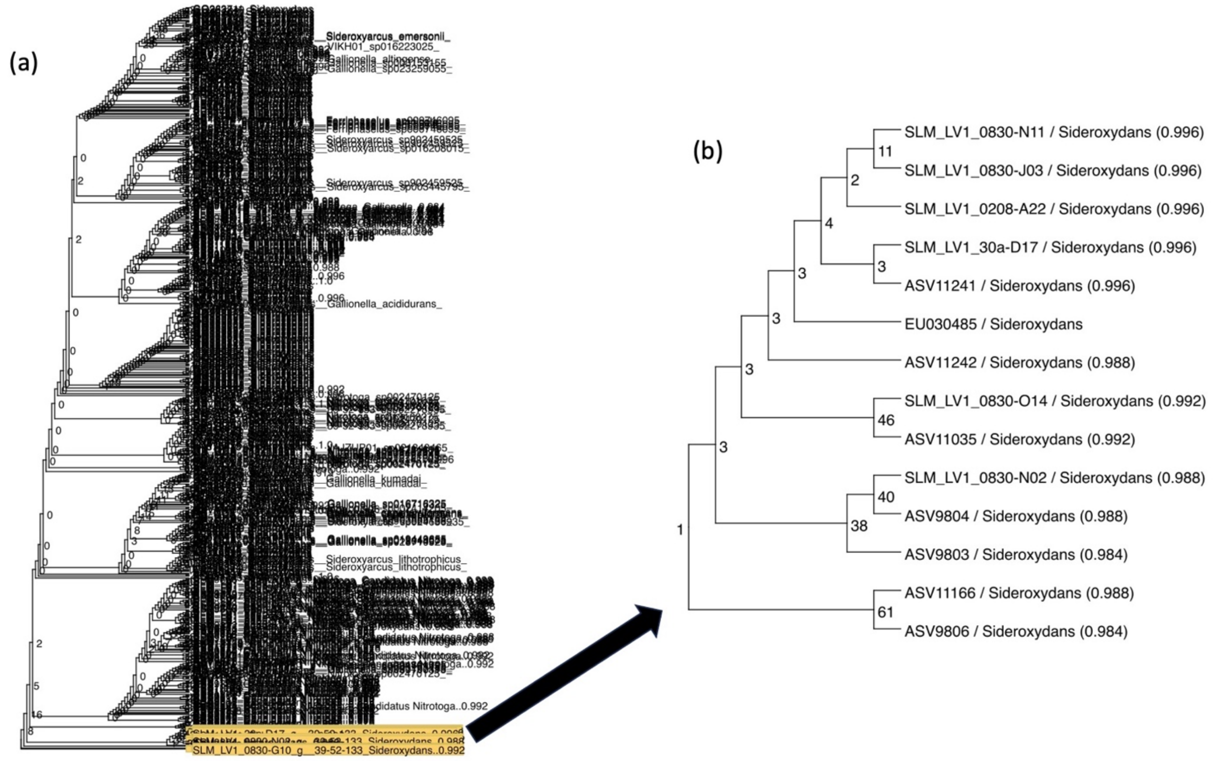


**Supplementary Fig. 5. Taxonomic comparison of *Sideroxydans* between SAGs and AVSs.** (a) Maximum-likelihood tree was reconstructed using 16S rRNA V4 sequences derived from four datasets: SAGs affiliated with the family Gallionellaceae (64 sequences), GTDB reference genomes in Gallionellaceae (68 sequences), ASVs assigned to Gallionellaceae (143 sequences), and 332 *Sideroxydans* sequences from the SILVA v138 database. A cluster containing SAG taxa grouped with a SILVA *Sideroxydans* sequence was highlighted in yellow. (b) Subtree extracted from the highlighted region in panel (a). Terminal leaves indicate the strain names (or sequence accession) followed by the closest SILVA taxonomic assignments, separated by a slash. Values in parentheses indicate the sequence similarity between queries and their closest SILVA 16S rRNA sequences. Bootstrap values are shown next to the corresponding nodes. Source data are provided as a Source Data file.

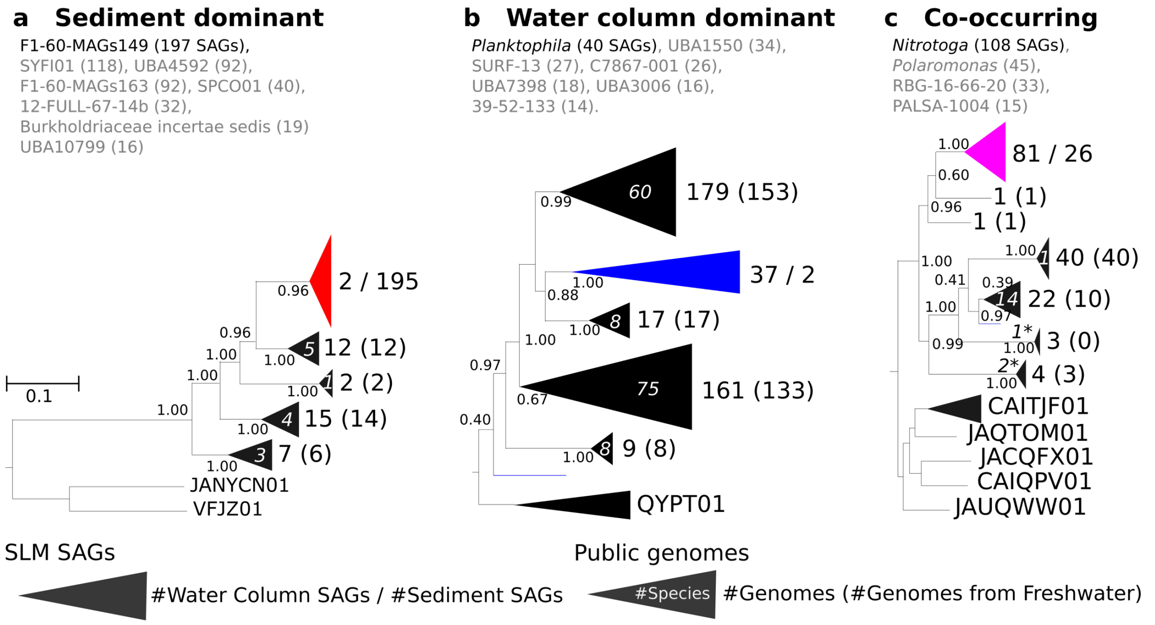


**Supplementary Fig. 6. Habitat preferences of the 19 most abundant genera.** The phylogenomic trees of SLM's SAGs belonging to the most abundant genera and their closely related public genomes (see Methods for detailed tree reconstruction). The trees were rooted by outgroup genomes that were chosen from the closest neighboring genera. The terminal taxa were collapsed into triangles, where height and width correlate with the number of genomes and phylogenetic depth, respectively. (**a**) Eight genera for which SAGs are dominant in sediments (red). (**b**) Seven genera for which SAGs are dominant in in the water column (blue), and (**C**) Four genera of SAGs found in both habitats that cluster together (magenta). The trees were visualized for F1-60-MAGs149 (**a**), *Planktophila* (**b**), and *Nitrotoga* (**c**) in bold that represent the largest number of SAGs. The Shimodaira-Hasegawa support values are shown next to the internal nodes.

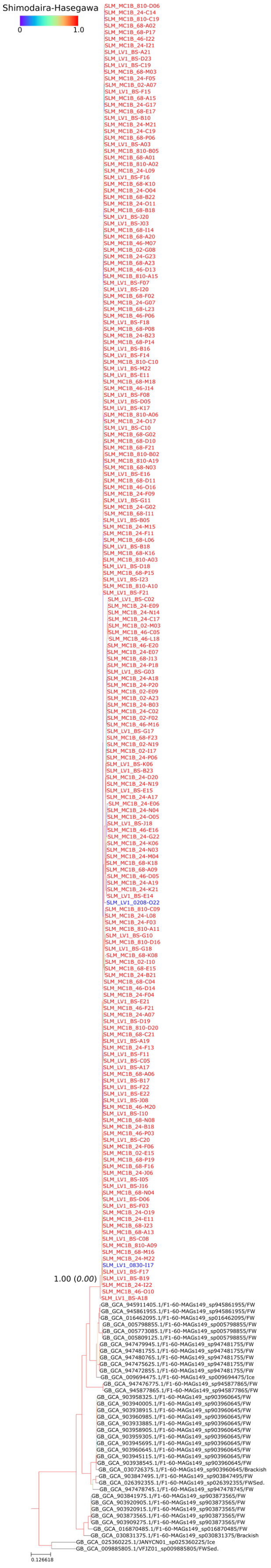
Shimodaira-Hasegawa
0
1.0
1.00 (0.00)
0.126618

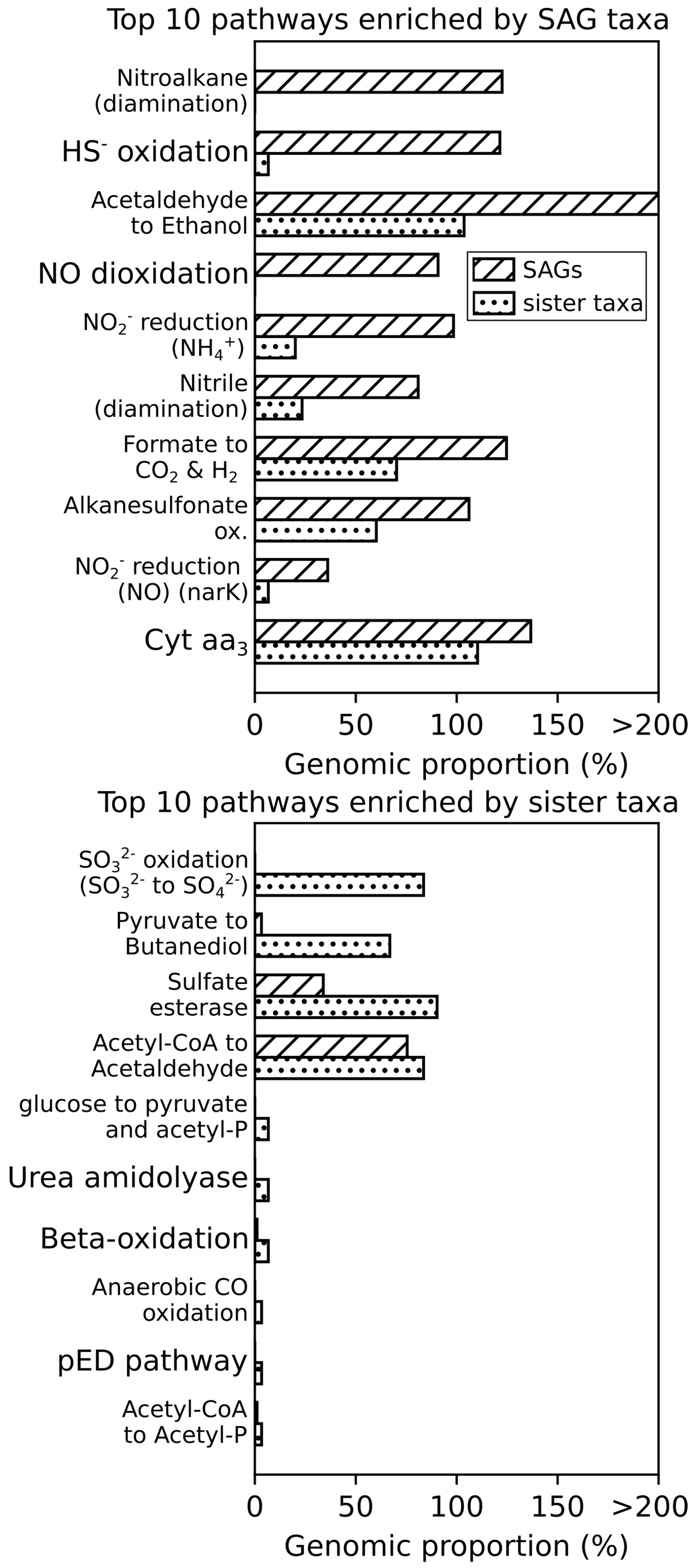
Top 10 pathways enriched by SAG taxa
Nitroalkane (diamination)
HS- oxidation
Acetaldehyde to Ethanol
NO dioxidation
NO2- reduction (NH4+)
Nitrile (diamination)
Formate to CO2 & H2
Alkanesulfonate ox.
NO2- reduction (NO) (narK)
Cyt aa3
SAGs
sister taxa
0
50
100
150
>200
Genomic proportion (%)
Top 10 pathways enriched by sister taxa
SO32- oxidation (SO32- to SO42-)
Pyruvate to Butanediol
Sulfate esterase
Acetyl-CoA to Acetaldehyde
glucose to pyruvate and acetyl-P
Urea amidolyase
Beta-oxidation
Anaerobic CO oxidation
pED pathway
Acetyl-CoA to Acetyl-P
0
50
100
150
>200
Genomic proportion (%)

**Supplementary Fig. 7. Phylogenomic trees of the genus F1-60-MAGs149.** Maximum-likelihood trees were reconstructed using the GTDB marker gene alignments of SAGs (lake water SAGs in blue, sedimentary SAGs in red, and SAGs from other taxonomic groups in green) and closely related GTDB genomes (taxa in black). Branch support (see color bar in the upper left) was assessed using SH values, ranging from blue (weak) to green (moderate) to red (strong). The value next to the branch of the common ancestor of the SAGs represents the genealogical sorting index, ranging from 0 (weakest) to 1 (strongest), which quantifies the strength of SAGs' monophyly. The value in parentheses denotes the P-value of the GSI. In the right panel, the upper graph shows the top 10 metabolic pathways enriched by SLM SAGs, while the lower graph shows the top 10 metabolic pathways enriched by sister taxa. Source data are provided as a Source Data file.

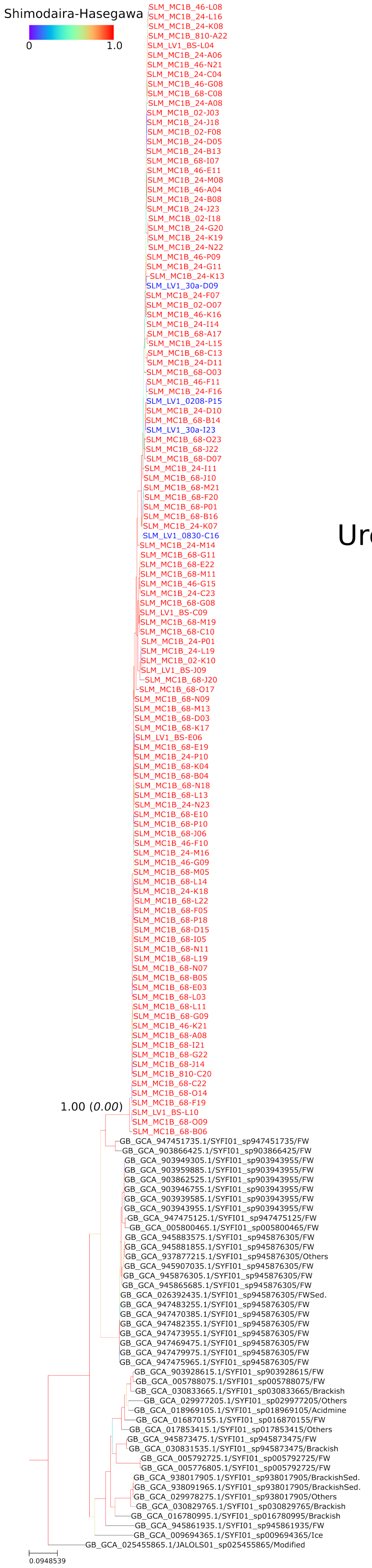

Shimodaira-Hasegawa
0
1.0
1.00 (0.00)
0.0948539


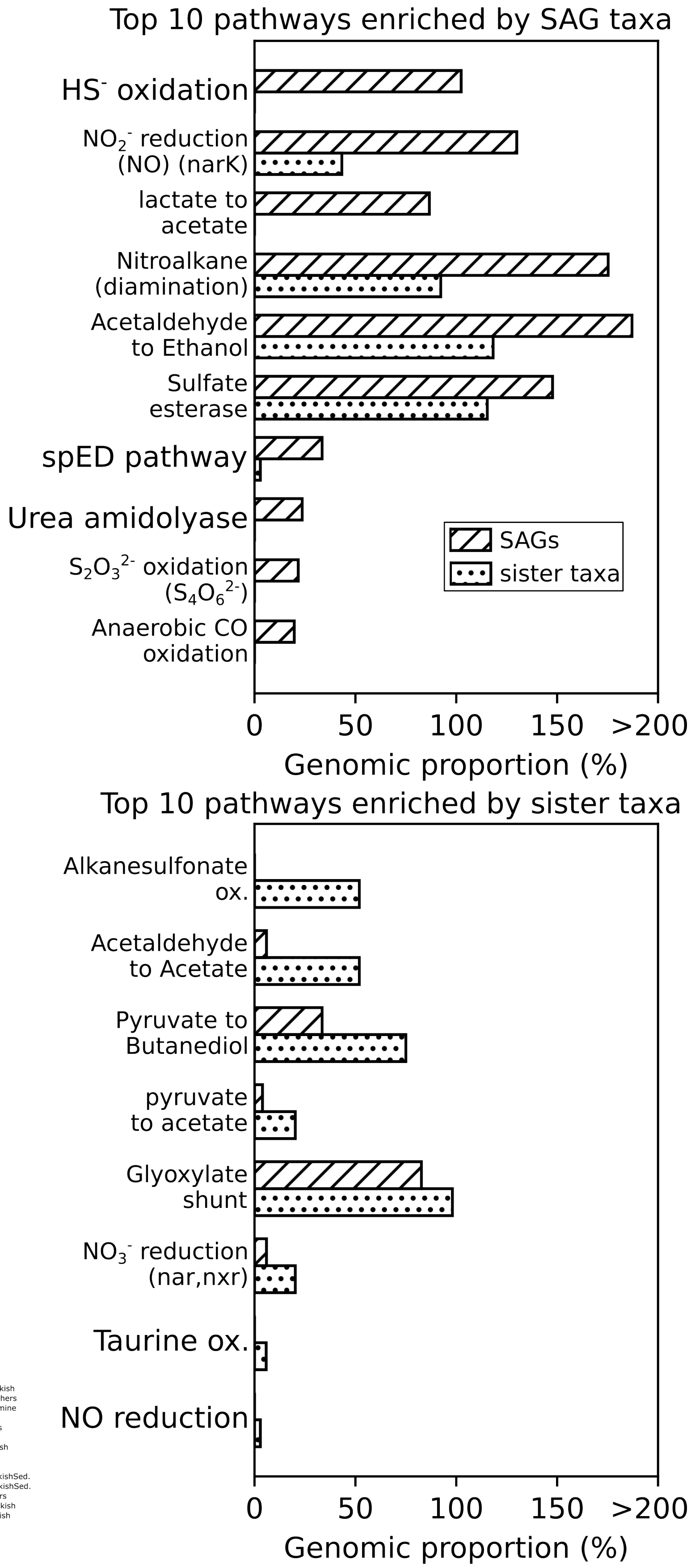

Top 10 pathways enriched by SAG taxa
HS$^-$ oxidation
NO$_2^-$ reduction (NO) (narK)
lactate to acetate
Nitroalkane (diamination)
Acetaldehyde to Ethanol
Sulfate esterase
spED pathway
Urea amidolyase
S$_2$O$_3^{2-}$ oxidation (S$_4$O$_6^{2-}$)
Anaerobic CO oxidation
SAGs
sister taxa
0
50
100
150
>200
Genomic proportion (%)
Top 10 pathways enriched by sister taxa
Alkanesulfonate ox.
Acetaldehyde to Acetate
Pyruvate to Butanediol
pyruvate to acetate
Glyoxylate shunt
NO$_3^-$ reduction (nar,nxr)
Taurine ox.
NO reduction
0
50
100
150
>200
Genomic proportion (%)

**Supplementary Fig. 8. Phylogenomic trees of the genus SYFI01.** Maximum-likelihood trees were reconstructed using the GTDB marker gene alignments of SAGs (lake water SAGs in blue, sedimentary SAGs in red, and SAGs from other taxonomic groups in green) and closely related GTDB genomes (taxa in black). Branch support (see color bar in the upper left) was assessed using SH values, ranging from blue (weak) to green (moderate) to red (strong). The value next to the branch of the common ancestor of the SAGs represents the genealogical sorting index, ranging from 0 (weakest) to 1 (strongest), which quantifies the strength of SAGs' monophyly. The value in parentheses denotes the P-value of the GSI. In the right panel, the upper graph shows the top 10 metabolic pathways enriched by SLM SAGs, while the lower graph shows the top 10 metabolic pathways enriched by sister taxa. Source data are provided as a Source Data file.

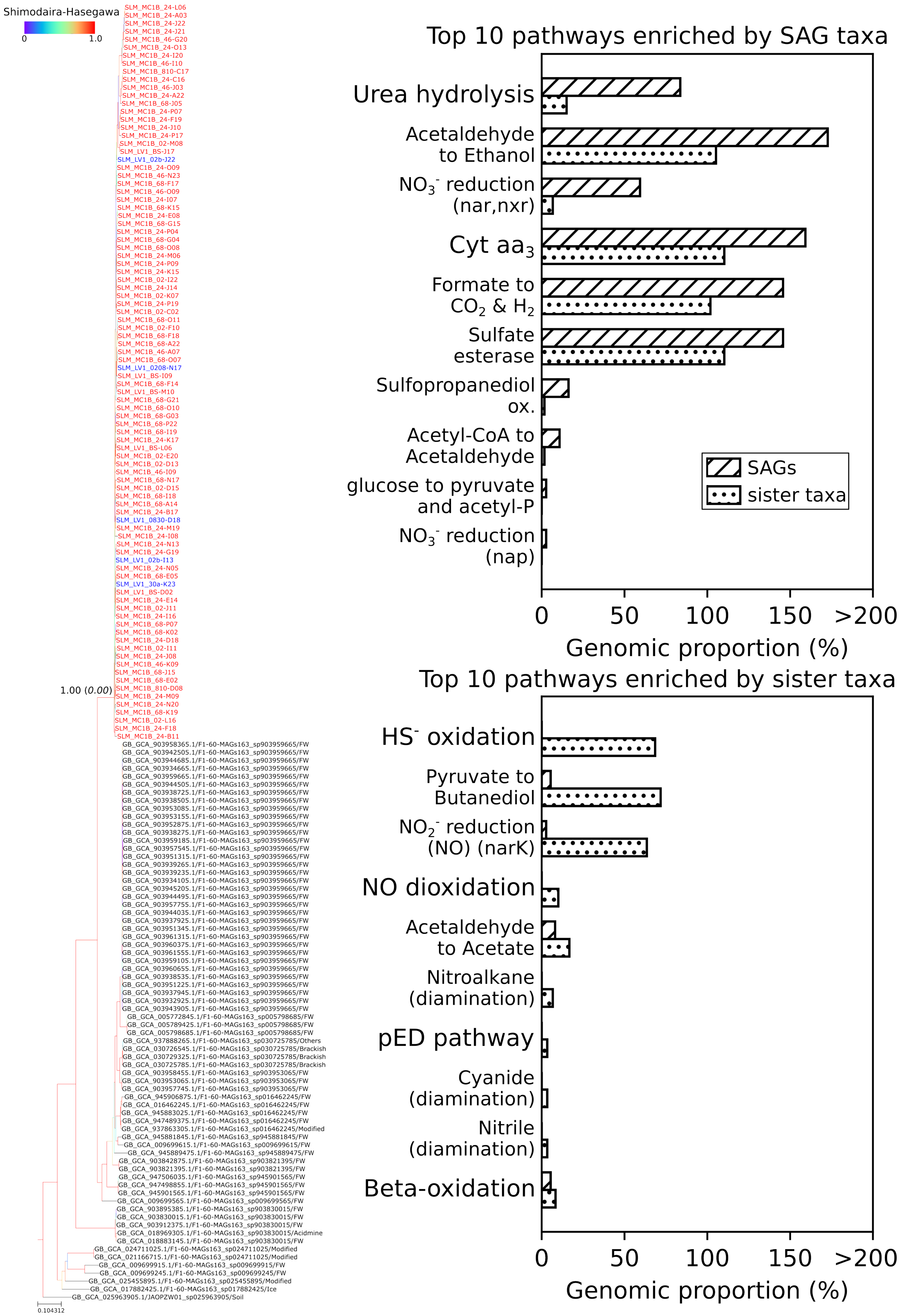
Shimodaira-Hasegawa
0
1.0
1.00 (0.00)
0.104312
Top 10 pathways enriched by SAG taxa
Urea hydrolysis
Acetaldehyde to Ethanol
$NO_3^-$ reduction (nar,nxr)
Cyt $aa_3$
Formate to $CO_2$ & $H_2$
Sulfate esterase
Sulfopropanediol ox.
Acetyl-CoA to Acetaldehyde
glucose to pyruvate and acetyl-P
$NO_3^-$ reduction (nap)
SAGs
sister taxa
0
50
100
150
>200
Genomic proportion (%)
Top 10 pathways enriched by sister taxa
$HS^-$ oxidation
Pyruvate to Butanediol
$NO_2^-$ reduction (NO) (narK)
NO dioxidation
Acetaldehyde to Acetate
Nitroalkane (diamination)
pED pathway
Cyanide (diamination)
Nitrile (diamination)
Beta-oxidation
0
50
100
150
>200
Genomic proportion (%)

**Supplementary Fig. 9. Phylogenomic trees of the genus F1-60-MAGs163.** Maximum-likelihood trees were reconstructed using the GTDB marker gene alignments of SAGs (lake water SAGs in blue, sedimentary SAGs in red, and SAGs from other taxonomic groups in green) and closely related GTDB genomes (taxa in black). Branch support (see color bar in the upper left) was assessed using SH values, ranging from blue (weak) to green (moderate) to red (strong). The value next to the branch of the common ancestor of the SAGs represents the genealogical sorting index, ranging from 0 (weakest) to 1 (strongest), which quantifies the strength of SAGs' monophyly. The value in parentheses denotes the P-value of the GSI. In the right panel, the upper graph shows the top 10 metabolic pathways enriched by SLM SAGs, while the lower graph shows the top 10 metabolic pathways enriched by sister taxa. Source data are provided as a Source Data file.

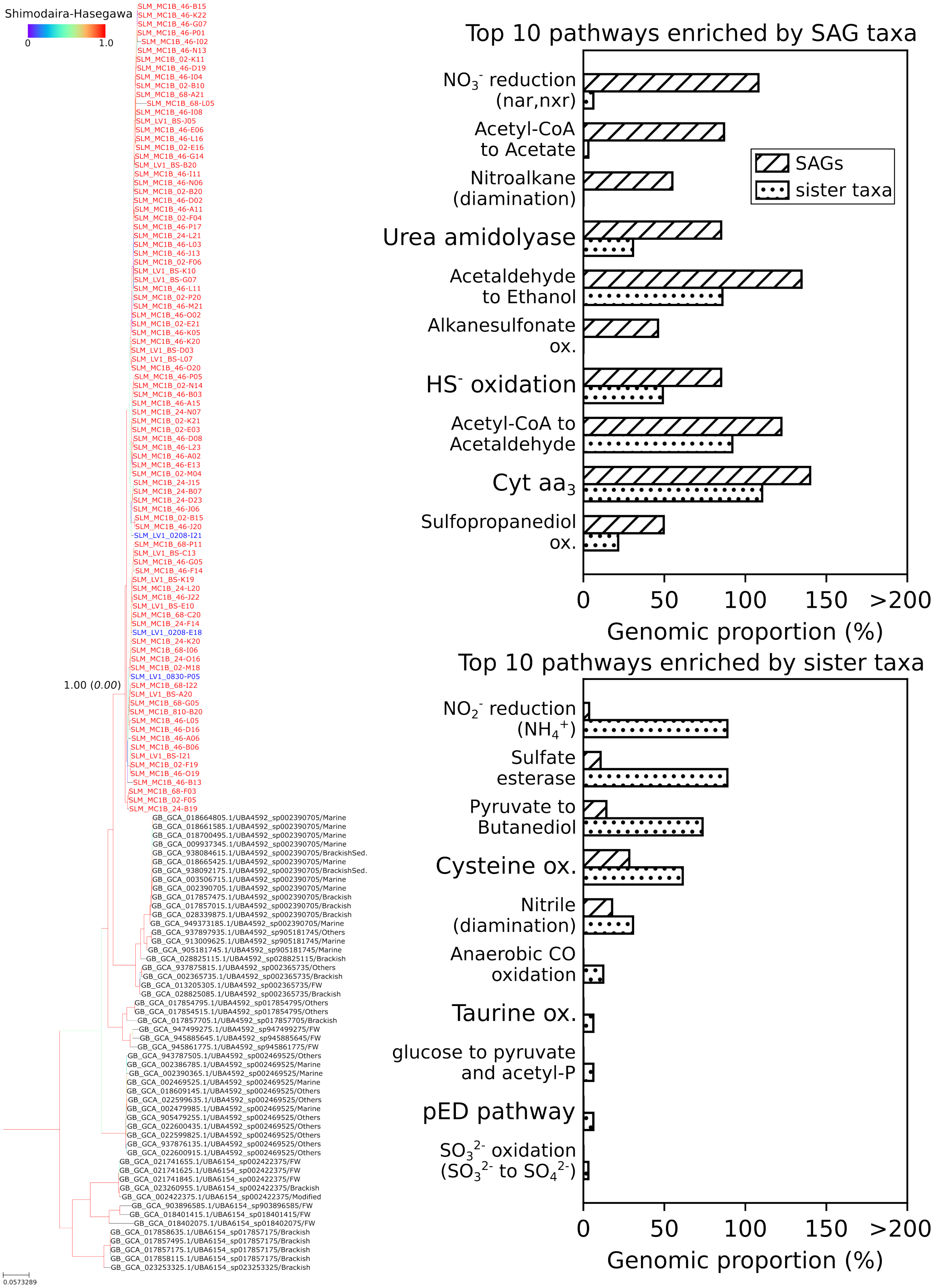


**Supplementary Fig. 10. Phylogenomic trees of the genus UBA4592.** Maximum-likelihood trees were reconstructed using the GTDB marker gene alignments of SAGs (lake water SAGs

in blue, sedimentary SAGs in red, and SAGs from other taxonomic groups in green) and closely related GTDB genomes (taxa in black). Branch support (see color bar in the upper left) was assessed using SH values, ranging from blue (weak) to green (moderate) to red (strong). The value next to the branch of the common ancestor of the SAGs represents the genealogical sorting index, ranging from 0 (weakest) to 1 (strongest), which quantifies the strength of SAGs' monophyly. The value in parentheses denotes the P-value of the GSI. In the right panel, the upper graph shows the top 10 metabolic pathways enriched by SLM SAGs, while the lower graph shows the top 10 metabolic pathways enriched by sister taxa. Source data are provided as a Source Data file.

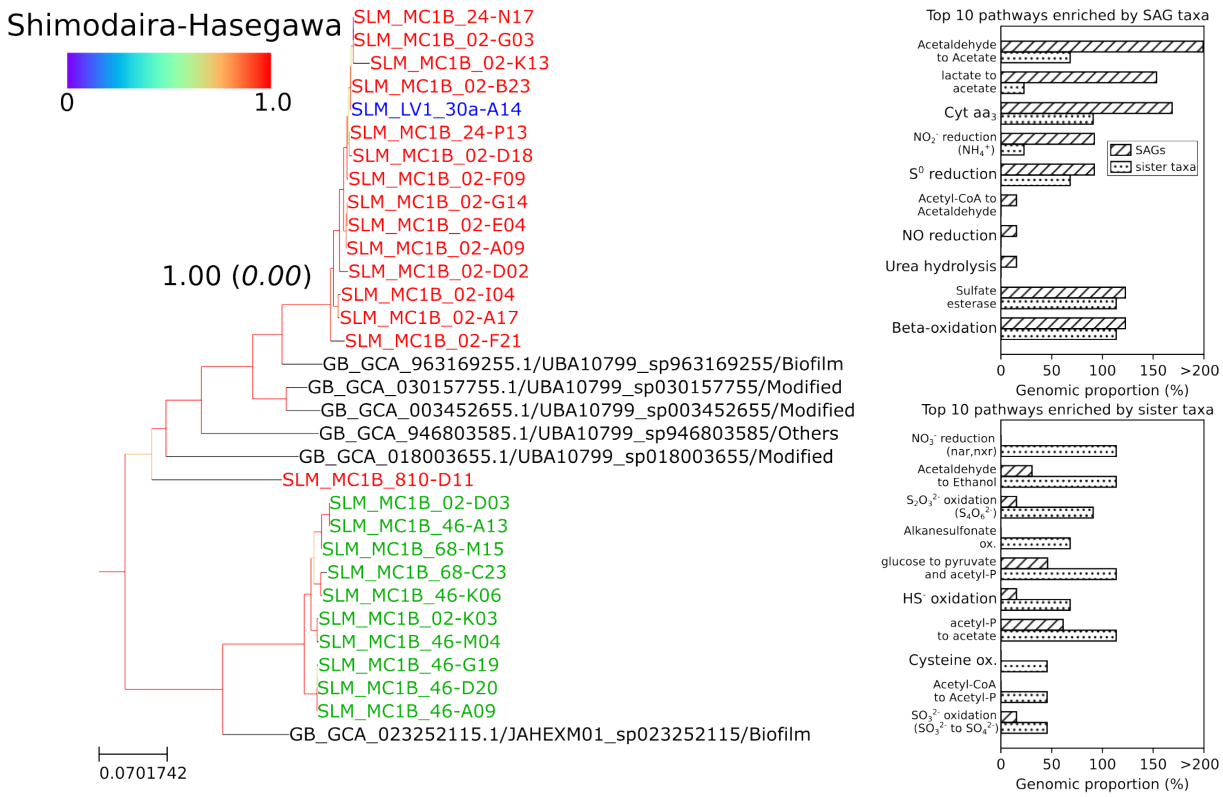


**Supplementary Fig.11. Phylogenomic trees of the genus UBA10799.** Maximum-likelihood trees were reconstructed using the GTDB marker gene alignments of SAGs (lake water SAGs in blue, sedimentary SAGs in red, and SAGs from other taxonomic groups in green) and closely related GTDB genomes (taxa in black). Branch support (see color bar in the upper left) was assessed using SH values, ranging from blue (weak) to green (moderate) to red (strong). The value next to the branch of the common ancestor of the SAGs represents the genealogical sorting index, ranging from 0 (weakest) to 1 (strongest), which quantifies the strength of SAGs' monophyly. The value in parentheses denotes the P-value of the GSI. In the right panel, the upper graph shows the top 10 metabolic pathways enriched by SLM SAGs, while the lower graph shows the top 10 metabolic pathways enriched by sister taxa. Source data are provided as a Source Data file.

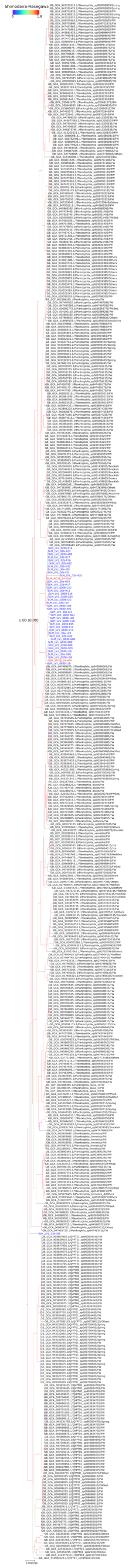

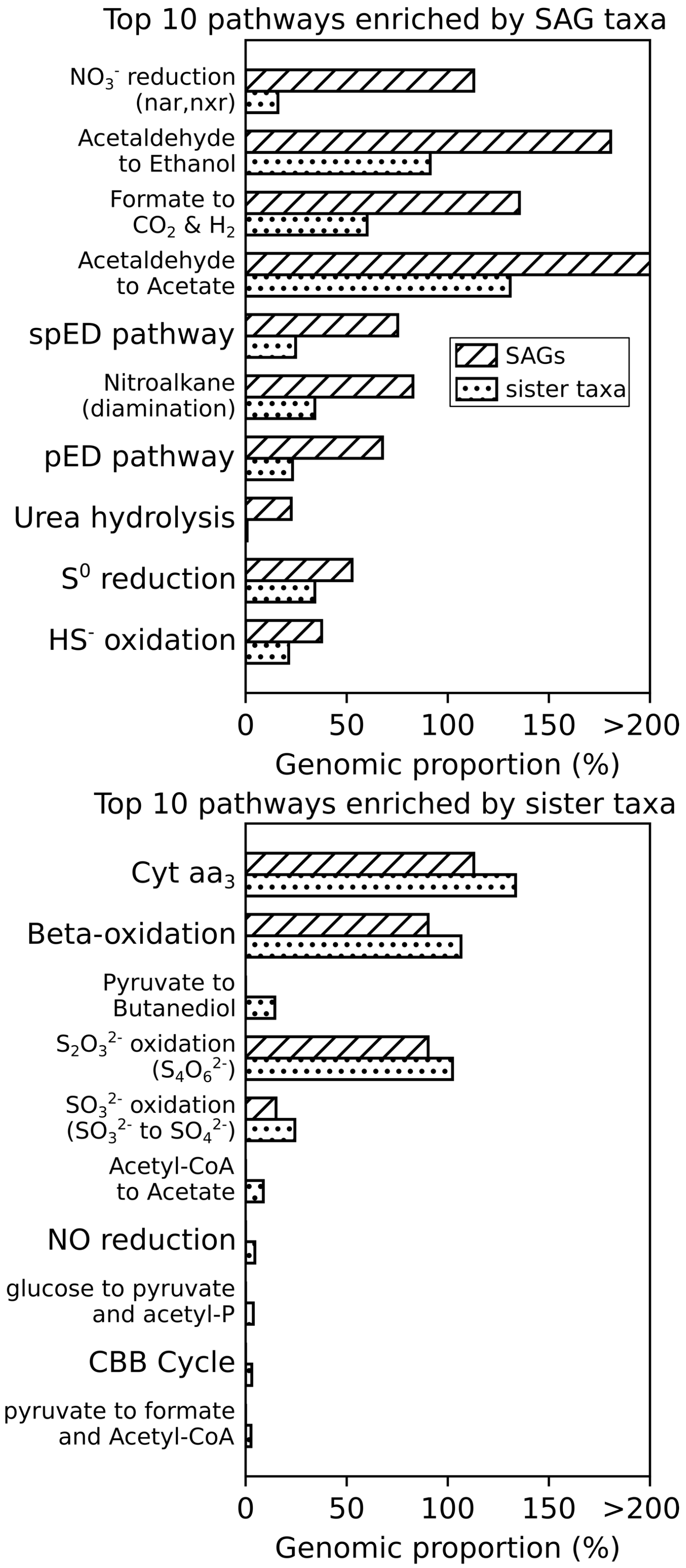

Top 10 pathways enriched by SAG taxa
$NO_3^-$ reduction (nar,nxr)
Acetaldehyde to Ethanol
Formate to $CO_2$ & $H_2$
Acetaldehyde to Acetate
spED pathway
Nitroalkane (diamination)
pED pathway
Urea hydrolysis
$S^0$ reduction
$HS^-$ oxidation
SAGs
sister taxa
0
50
100
150
>200
Genomic proportion (%)
Top 10 pathways enriched by sister taxa
Cyt $aa_3$
Beta-oxidation
Pyruvate to Butanediol
$S_2O_3^{2-}$ oxidation ($S_4O_6^{2-}$)
$SO_3^{2-}$ oxidation ($SO_3^{2-}$ to $SO_4^{2-}$)
Acetyl-CoA to Acetate
NO reduction
glucose to pyruvate and acetyl-P
CBB Cycle
pyruvate to formate and Acetyl-CoA
0
50
100
150
>200
Genomic proportion (%)

**Supplementary Fig. 12. Phylogenomic trees of the genus *Planktophila*.** Maximum-likelihood trees were reconstructed using the GTDB marker gene alignments of SAGs (lake water SAGs in blue, sedimentary SAGs in red, and SAGs from other taxonomic groups in green) and closely related GTDB genomes (taxa in black). Branch support (see color bar in the upper left) was assessed using SH values, ranging from blue (weak) to green (moderate) to red (strong). The value next to the branch of the common ancestor of the SAGs represents the genealogical sorting index, ranging from 0 (weakest) to 1 (strongest), which quantifies the strength of SAGs' monophyly. The value in parentheses denotes the P-value of the GSI. In the right panel, the upper graph shows the top 10 metabolic pathways enriched by SLM SAGs, while the lower graph shows the top 10 metabolic pathways enriched by sister taxa. Source data are provided as a Source Data file.

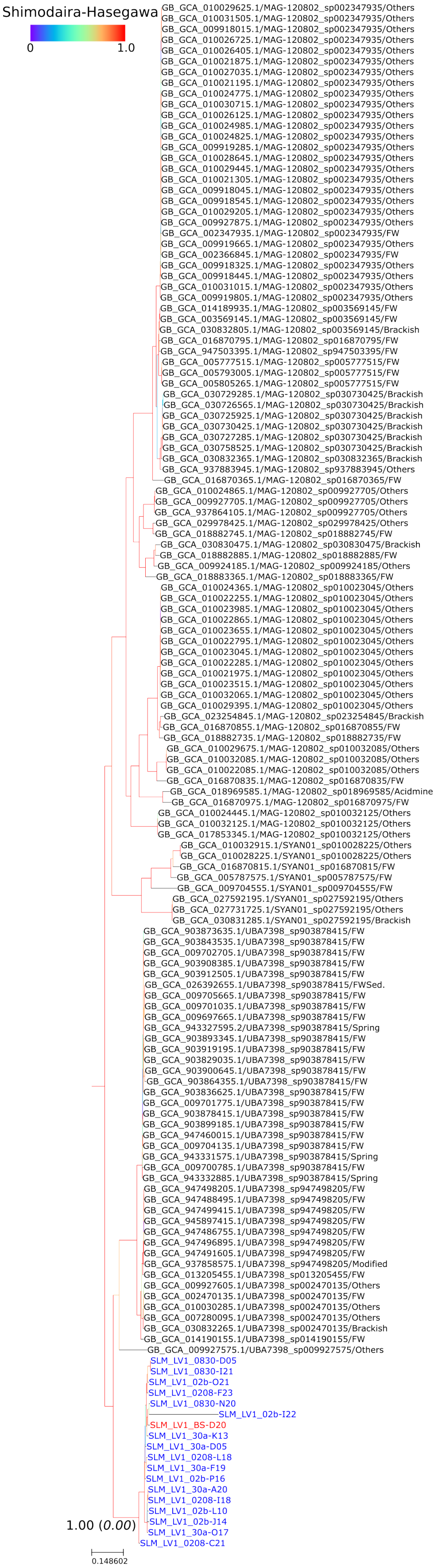

Shimodaira-Hasegawa
0
1.0
GB_GCA_010029625.1/MAG-120802_sp002347935/Others
GB_GCA_010031505.1/MAG-120802_sp002347935/Others
GB_GCA_009918015.1/MAG-120802_sp002347935/Others
GB_GCA_010026725.1/MAG-120802_sp002347935/Others
GB_GCA_010026405.1/MAG-120802_sp002347935/Others
GB_GCA_010021875.1/MAG-120802_sp002347935/Others
GB_GCA_010027035.1/MAG-120802_sp002347935/Others
GB_GCA_010021195.1/MAG-120802_sp002347935/Others
GB_GCA_010024775.1/MAG-120802_sp002347935/Others
GB_GCA_010030715.1/MAG-120802_sp002347935/Others
GB_GCA_010026125.1/MAG-120802_sp002347935/Others
GB_GCA_010024985.1/MAG-120802_sp002347935/Others
GB_GCA_010024825.1/MAG-120802_sp002347935/Others
GB_GCA_009919285.1/MAG-120802_sp002347935/Others
GB_GCA_010028645.1/MAG-120802_sp002347935/Others
GB_GCA_010029445.1/MAG-120802_sp002347935/Others
GB_GCA_010021305.1/MAG-120802_sp002347935/Others
GB_GCA_009918045.1/MAG-120802_sp002347935/Others
GB_GCA_009918545.1/MAG-120802_sp002347935/Others
GB_GCA_010029205.1/MAG-120802_sp002347935/Others
GB_GCA_009927875.1/MAG-120802_sp002347935/Others
GB_GCA_002347935.1/MAG-120802_sp002347935/FW
GB_GCA_009919665.1/MAG-120802_sp002347935/Others
GB_GCA_002366845.1/MAG-120802_sp002347935/FW
GB_GCA_009918325.1/MAG-120802_sp002347935/Others
GB_GCA_009918445.1/MAG-120802_sp002347935/Others
GB_GCA_010031015.1/MAG-120802_sp002347935/Others
GB_GCA_009919805.1/MAG-120802_sp002347935/Others
GB_GCA_014189935.1/MAG-120802_sp003569145/FW
GB_GCA_003569145.1/MAG-120802_sp003569145/FW
GB_GCA_030832805.1/MAG-120802_sp003569145/Brackish
GB_GCA_016870795.1/MAG-120802_sp016870795/FW
GB_GCA_947503395.1/MAG-120802_sp947503395/FW
GB_GCA_005777515.1/MAG-120802_sp005777515/FW
GB_GCA_005793005.1/MAG-120802_sp005777515/FW
GB_GCA_005805265.1/MAG-120802_sp005777515/FW
GB_GCA_030729285.1/MAG-120802_sp030730425/Brackish
GB_GCA_030726565.1/MAG-120802_sp030730425/Brackish
GB_GCA_030725925.1/MAG-120802_sp030730425/Brackish
GB_GCA_030730425.1/MAG-120802_sp030730425/Brackish
GB_GCA_030727285.1/MAG-120802_sp030730425/Brackish
GB_GCA_030758525.1/MAG-120802_sp030730425/Brackish
GB_GCA_030832365.1/MAG-120802_sp030832365/Brackish
GB_GCA_937883945.1/MAG-120802_sp937883945/Others
GB_GCA_016870365.1/MAG-120802_sp016870365/FW
GB_GCA_010024865.1/MAG-120802_sp009927705/Others
GB_GCA_009927705.1/MAG-120802_sp009927705/Others
GB_GCA_937864105.1/MAG-120802_sp009927705/Others
GB_GCA_029978425.1/MAG-120802_sp029978425/Others
GB_GCA_018882745.1/MAG-120802_sp018882745/FW
GB_GCA_030830475.1/MAG-120802_sp030830475/Brackish
GB_GCA_018882885.1/MAG-120802_sp018882885/FW
GB_GCA_009924185.1/MAG-120802_sp009924185/Others
GB_GCA_018883365.1/MAG-120802_sp018883365/FW
GB_GCA_010024365.1/MAG-120802_sp010023045/Others
GB_GCA_010022255.1/MAG-120802_sp010023045/Others
GB_GCA_010023985.1/MAG-120802_sp010023045/Others
GB_GCA_010022865.1/MAG-120802_sp010023045/Others
GB_GCA_010023655.1/MAG-120802_sp010023045/Others
GB_GCA_010022795.1/MAG-120802_sp010023045/Others
GB_GCA_010023045.1/MAG-120802_sp010023045/Others
GB_GCA_010022285.1/MAG-120802_sp010023045/Others
GB_GCA_010021975.1/MAG-120802_sp010023045/Others
GB_GCA_010023515.1/MAG-120802_sp010023045/Others
GB_GCA_010032065.1/MAG-120802_sp010023045/Others
GB_GCA_010029395.1/MAG-120802_sp010023045/Others
GB_GCA_023254845.1/MAG-120802_sp023254845/Brackish
GB_GCA_016870855.1/MAG-120802_sp016870855/FW
GB_GCA_018882735.1/MAG-120802_sp018882735/FW
GB_GCA_010029675.1/MAG-120802_sp010032085/Others
GB_GCA_010032085.1/MAG-120802_sp010032085/Others
GB_GCA_010022085.1/MAG-120802_sp010032085/Others
GB_GCA_016870835.1/MAG-120802_sp016870835/FW
GB_GCA_018969585.1/MAG-120802_sp018969585/Acidmine
GB_GCA_016870975.1/MAG-120802_sp016870975/FW
GB_GCA_010024445.1/MAG-120802_sp010032125/Others
GB_GCA_010032125.1/MAG-120802_sp010032125/Others
GB_GCA_017853345.1/MAG-120802_sp010032125/Others
GB_GCA_010032915.1/SYAN01_sp010028225/Others
GB_GCA_010028225.1/SYAN01_sp010028225/Others
GB_GCA_016870815.1/SYAN01_sp016870815/FW
GB_GCA_005787575.1/SYAN01_sp005787575/FW
GB_GCA_009704555.1/SYAN01_sp009704555/FW
GB_GCA_027592195.1/SYAN01_sp027592195/Others
GB_GCA_027731725.1/SYAN01_sp027592195/Others
GB_GCA_030831285.1/SYAN01_sp027592195/Brackish
GB_GCA_903873635.1/UBA7398_sp903878415/FW
GB_GCA_903843535.1/UBA7398_sp903878415/FW
GB_GCA_009702705.1/UBA7398_sp903878415/FW
GB_GCA_903908385.1/UBA7398_sp903878415/FW
GB_GCA_903912505.1/UBA7398_sp903878415/FW
GB_GCA_026392655.1/UBA7398_sp903878415/FWSed.
GB_GCA_009705665.1/UBA7398_sp903878415/FW
GB_GCA_009701035.1/UBA7398_sp903878415/FW
GB_GCA_009697665.1/UBA7398_sp903878415/FW
GB_GCA_943327595.2/UBA7398_sp903878415/Spring
GB_GCA_903893345.1/UBA7398_sp903878415/FW
GB_GCA_903919195.1/UBA7398_sp903878415/FW
GB_GCA_903829035.1/UBA7398_sp903878415/FW
GB_GCA_903900645.1/UBA7398_sp903878415/FW
GB_GCA_903864355.1/UBA7398_sp903878415/FW
GB_GCA_903836625.1/UBA7398_sp903878415/FW
GB_GCA_009701775.1/UBA7398_sp903878415/FW
GB_GCA_903878415.1/UBA7398_sp903878415/FW
GB_GCA_903899185.1/UBA7398_sp903878415/FW
GB_GCA_947460015.1/UBA7398_sp903878415/FW
GB_GCA_009704135.1/UBA7398_sp903878415/FW
GB_GCA_943331575.1/UBA7398_sp903878415/Spring
GB_GCA_009700785.1/UBA7398_sp903878415/FW
GB_GCA_943332885.1/UBA7398_sp903878415/Spring
GB_GCA_947498205.1/UBA7398_sp947498205/FW
GB_GCA_947488495.1/UBA7398_sp947498205/FW
GB_GCA_947499415.1/UBA7398_sp947498205/FW
GB_GCA_945897415.1/UBA7398_sp947498205/FW
GB_GCA_947486755.1/UBA7398_sp947498205/FW
GB_GCA_947496895.1/UBA7398_sp947498205/FW
GB_GCA_947491605.1/UBA7398_sp947498205/FW
GB_GCA_937858575.1/UBA7398_sp947498205/Modified
GB_GCA_013205455.1/UBA7398_sp013205455/FW
GB_GCA_009927605.1/UBA7398_sp002470135/Others
GB_GCA_002470135.1/UBA7398_sp002470135/FW
GB_GCA_010030285.1/UBA7398_sp002470135/Others
GB_GCA_007280095.1/UBA7398_sp002470135/Others
GB_GCA_030832265.1/UBA7398_sp002470135/Brackish
GB_GCA_014190155.1/UBA7398_sp014190155/FW
GB_GCA_009927575.1/UBA7398_sp009927575/Others
SLM_LV1_0830-D05
SLM_LV1_0830-I21
SLM_LV1_02b-O21
SLM_LV1_0208-F23
SLM_LV1_0830-N20
SLM_LV1_02b-I22
SLM_LV1_BS-D20
SLM_LV1_30a-K13
SLM_LV1_30a-D05
SLM_LV1_0208-L18
SLM_LV1_30a-F19
SLM_LV1_02b-P16
SLM_LV1_30a-A20
SLM_LV1_0208-I18
SLM_LV1_02b-L10
SLM_LV1_02b-J14
SLM_LV1_30a-O17
SLM_LV1_0208-C21
1.00 (0.00)
0.148602


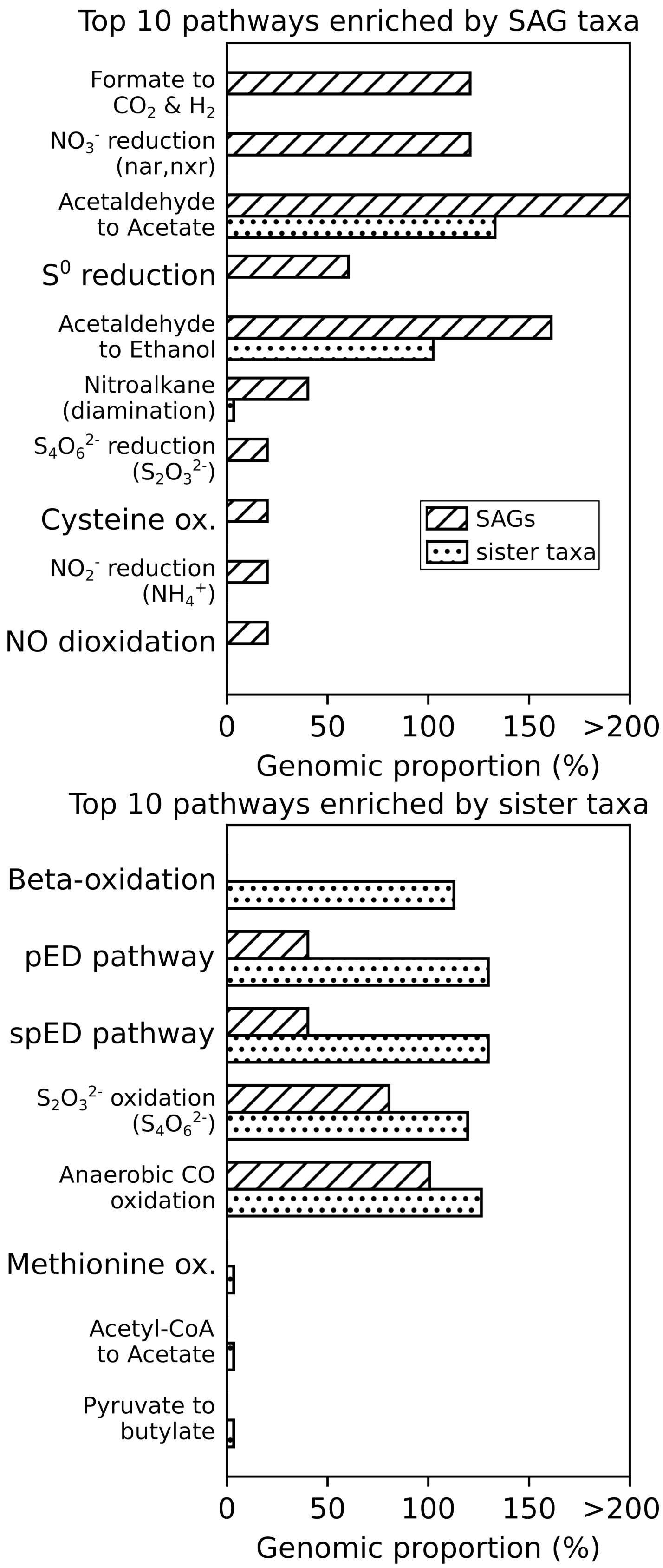

Top 10 pathways enriched by SAG taxa
Formate to CO2 & H2
NO3- reduction (nar,nxr)
Acetaldehyde to Acetate
S0 reduction
Acetaldehyde to Ethanol
Nitroalkane (diamination)
S4O62- reduction (S2O32-)
Cysteine ox.
NO2- reduction (NH4+)
NO dioxidation
SAGs
sister taxa
0
50
100
150
>200
Genomic proportion (%)
Top 10 pathways enriched by sister taxa
Beta-oxidation
pED pathway
spED pathway
S2O32- oxidation (S4O62-)
Anaerobic CO oxidation
Methionine ox.
Acetyl-CoA to Acetate
Pyruvate to butylate
0
50
100
150
>200
Genomic proportion (%)

**Supplementary Fig. 13. Phylogenomic trees of the genus UBA7398.** Maximum-likelihood trees were reconstructed using the GTDB marker gene alignments of SAGs (lake water SAGs in blue, sedimentary SAGs in red, and SAGs from other taxonomic groups in green) and closely related GTDB genomes (taxa in black). Branch support (see color bar in the upper left) was assessed using SH values, ranging from blue (weak) to green (moderate) to red (strong). The value next to the branch of the common ancestor of the SAGs represents the genealogical sorting index, ranging from 0 (weakest) to 1 (strongest), which quantifies the strength of SAGs' monophyly. The value in parentheses denotes the P-value of the GSI. In the right panel, the upper graph shows the top 10 metabolic pathways enriched by SLM SAGs, while the lower graph shows the top 10 metabolic pathways enriched by sister taxa. Source data are provided as a Source Data file.

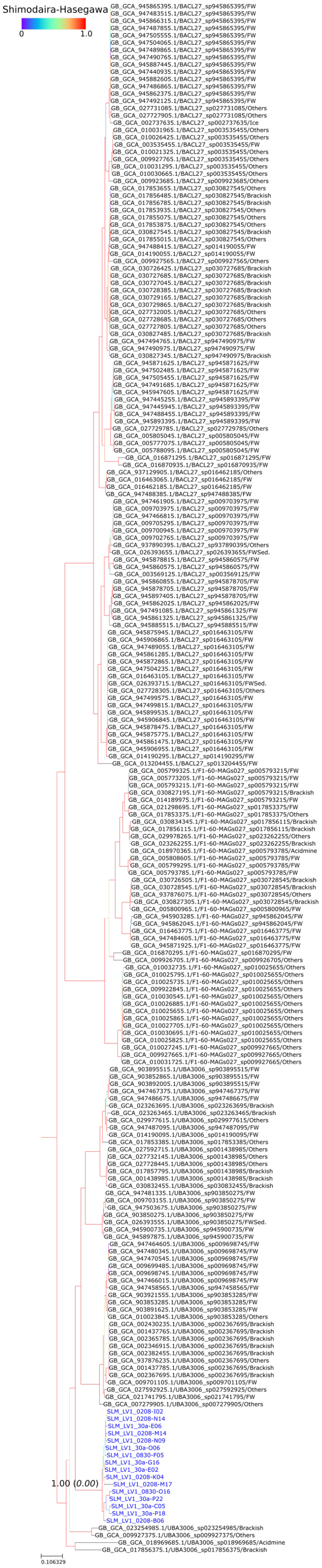
Shimodaira-Hasegawa
0
1.0
1.00 (0.00)
0.106329

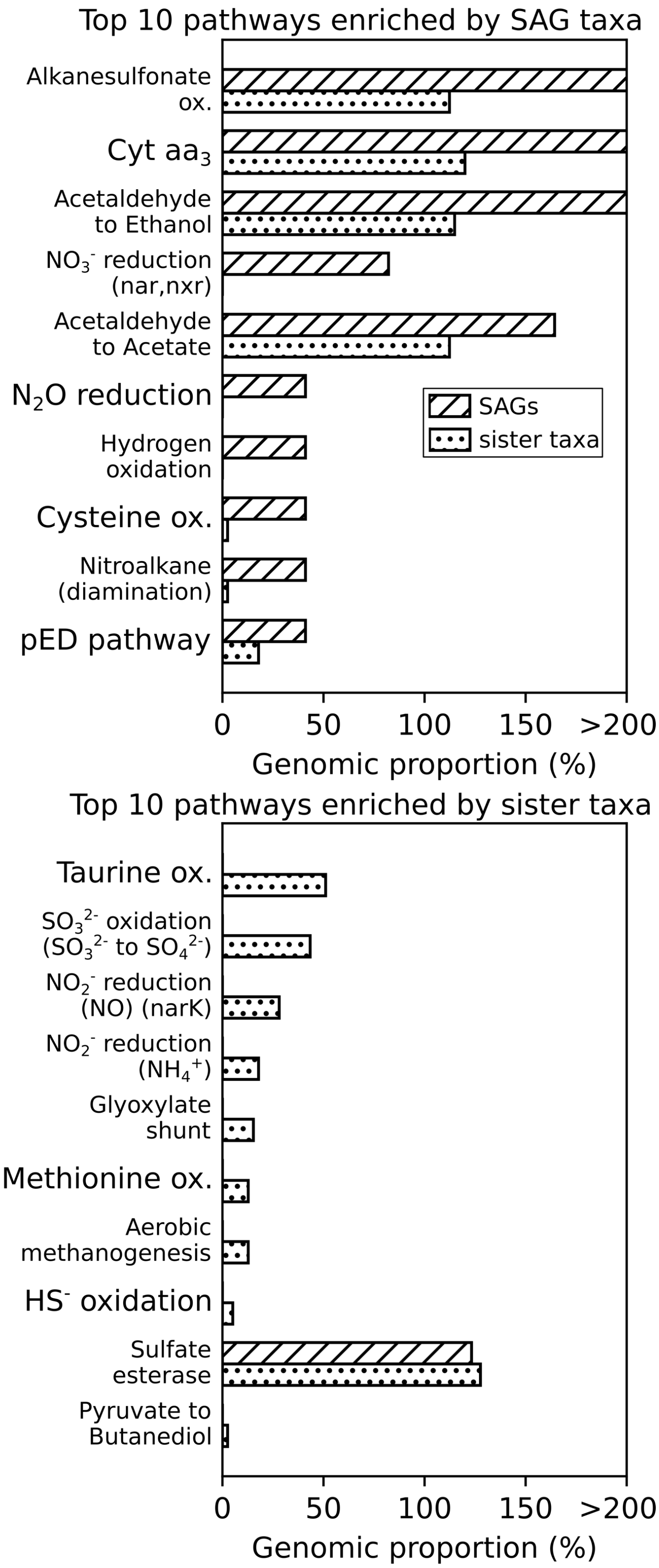
Top 10 pathways enriched by SAG taxa
Alkanesulfonate ox.
Cyt $aa_3$
Acetaldehyde to Ethanol
$NO_3^-$ reduction (nar,nxr)
Acetaldehyde to Acetate
$N_2O$ reduction
Hydrogen oxidation
Cysteine ox.
Nitroalkane (diamination)
pED pathway
SAGs
sister taxa
0
50
100
150
>200
Genomic proportion (%)
Top 10 pathways enriched by sister taxa
Taurine ox.
$SO_3^{2-}$ oxidation ($SO_3^{2-}$ to $SO_4^{2-}$)
$NO_2^-$ reduction (NO) (narK)
$NO_2^-$ reduction ($NH_4^+$)
Glyoxylate shunt
Methionine ox.
Aerobic methanogenesis
$HS^-$ oxidation
Sulfate esterase
Pyruvate to Butanediol
0
50
100
150
>200
Genomic proportion (%)

**Supplementary Fig. 14. Phylogenomic trees of the genus UBA3006.** Maximum-likelihood trees were reconstructed using the GTDB marker gene alignments of SAGs (lake water SAGs in blue, sedimentary SAGs in red, and SAGs from other taxonomic groups in green) and closely related GTDB genomes (taxa in black). Branch support (see color bar in the upper left) was assessed using SH values, ranging from blue (weak) to green (moderate) to red (strong). The value next to the branch of the common ancestor of the SAGs represents the genealogical sorting index, ranging from 0 (weakest) to 1 (strongest), which quantifies the strength of SAGs' monophyly. The value in parentheses denotes the P-value of the GSI. In the right panel, the upper graph shows the top 10 metabolic pathways enriched by SLM SAGs, while the lower graph shows the top 10 metabolic pathways enriched by sister taxa. Source data are provided as a Source Data file.

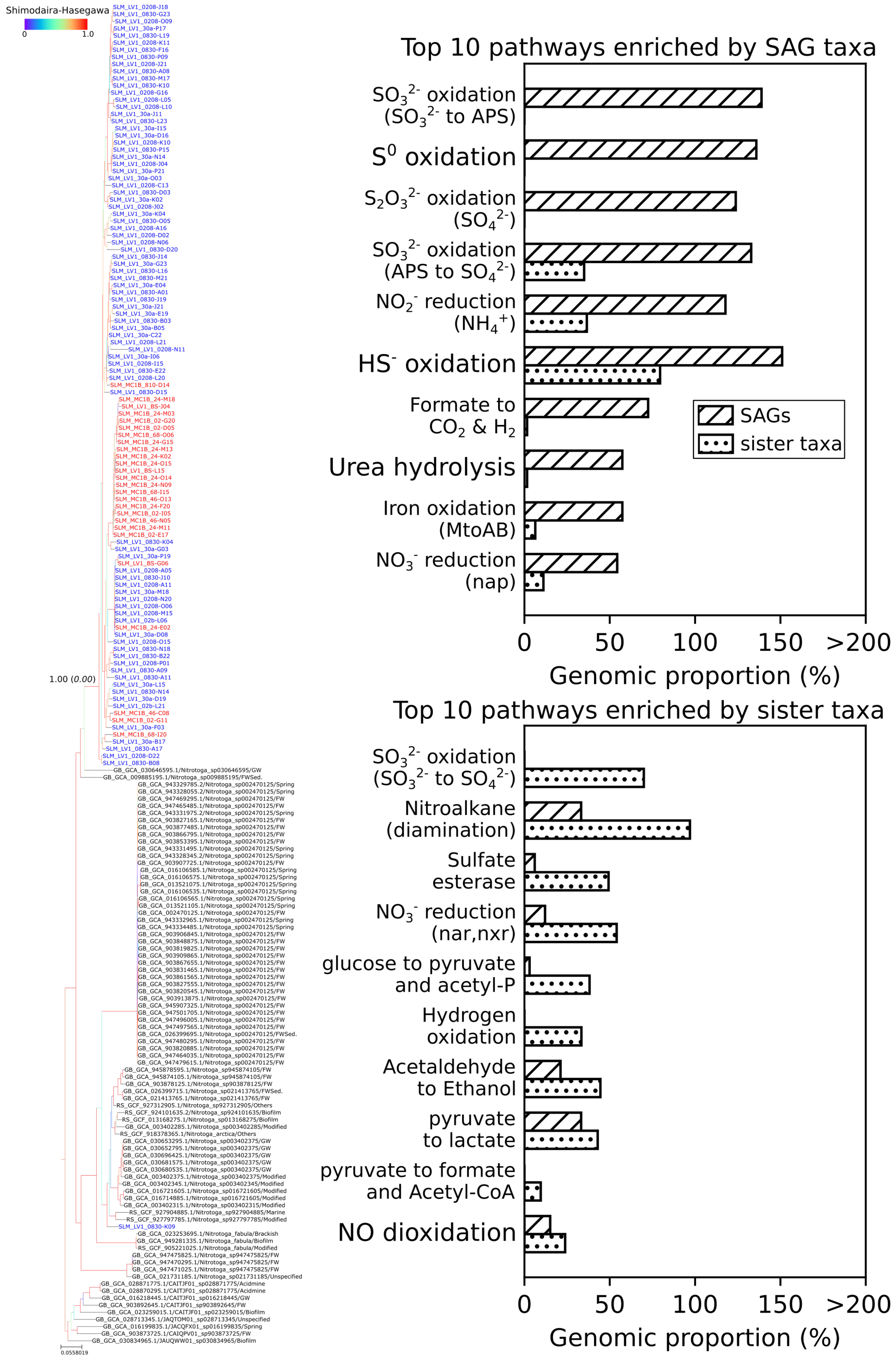

Shimodaira-Hasegawa
0
1.0
1.00 (0.00)
Top 10 pathways enriched by SAG taxa
SO3 2- oxidation (SO3 2- to APS)
S0 oxidation
S2O3 2- oxidation (SO4 2-)
SO3 2- oxidation (APS to SO4 2-)
NO2 - reduction (NH4 +)
HS- oxidation
Formate to CO2 & H2
Urea hydrolysis
Iron oxidation (MtoAB)
NO3 - reduction (nap)
SAGs
sister taxa
0
50
100
150
>200
Genomic proportion (%)
Top 10 pathways enriched by sister taxa
SO3 2- oxidation (SO3 2- to SO4 2-)
Nitroalkane (diamination)
Sulfate esterase
NO3 - reduction (nar,nxr)
glucose to pyruvate and acetyl-P
Hydrogen oxidation
Acetaldehyde to Ethanol
pyruvate to lactate
pyruvate to formate and Acetyl-CoA
NO dioxidation
0
50
100
150
>200
Genomic proportion (%)

**Supplementary Fig. 15. Phylogenomic trees of the genus *Nitrotoga*.** Maximum-likelihood trees were reconstructed using the GTDB marker gene alignments of SAGs (lake water SAGs in blue, sedimentary SAGs in red, and SAGs from other taxonomic groups in green) and closely related GTDB genomes (taxa in black). Branch support (see color bar in the upper left) was assessed using SH values, ranging from blue (weak) to green (moderate) to red (strong). The value next to the branch of the common ancestor of the SAGs represents the genealogical sorting index, ranging from 0 (weakest) to 1 (strongest), which quantifies the strength of SAGs' monophyly. The value in parentheses denotes the P-value of the GSI. In the right panel, the upper graph shows the top 10 metabolic pathways enriched by SLM SAGs, while the lower graph shows the top 10 metabolic pathways enriched by sister taxa. Source data are provided as a Source Data file.

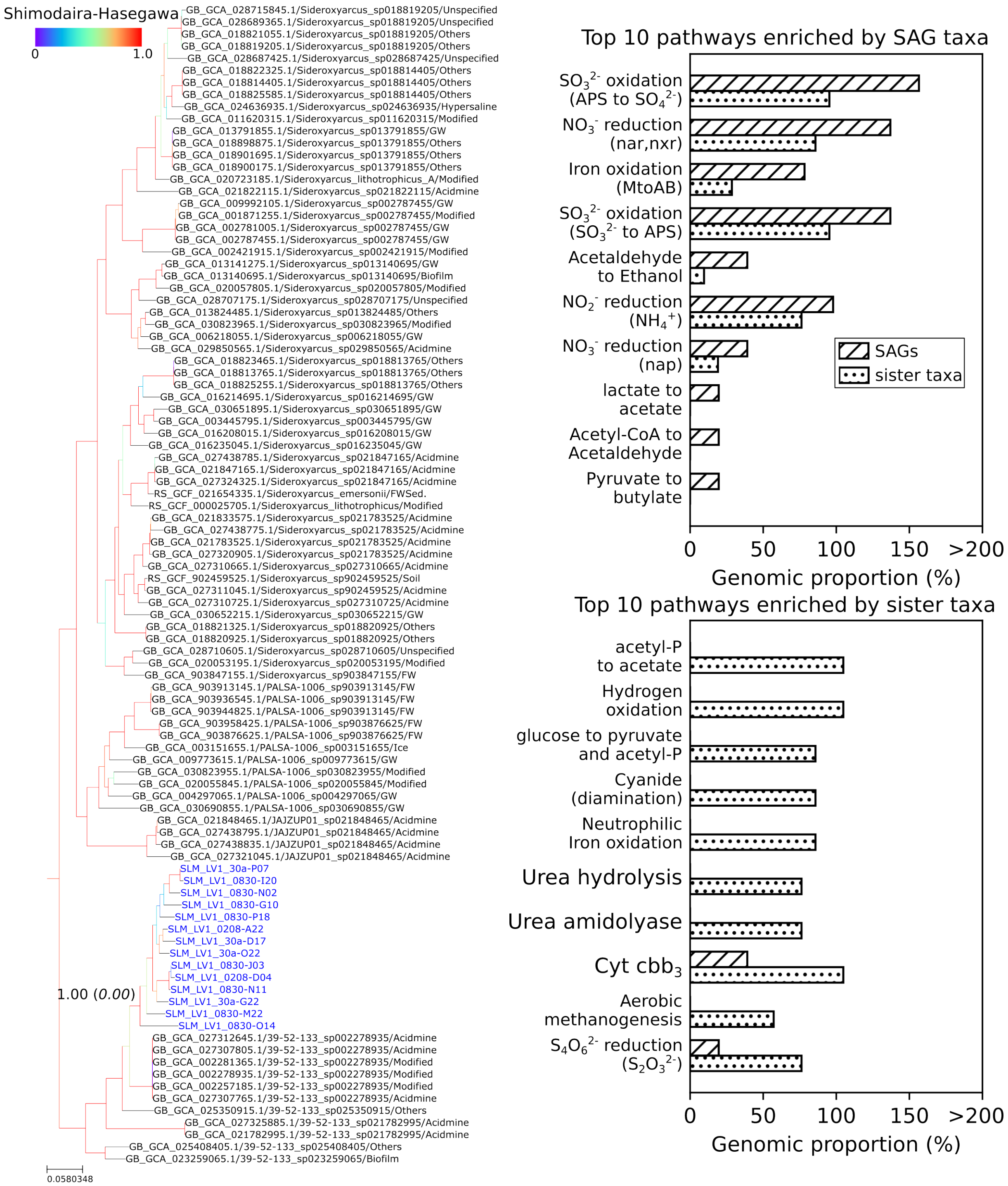


**Supplementary Fig. 16. Phylogenomic trees of the genus 39-52-133.** Maximum-likelihood trees were reconstructed using the GTDB marker gene alignments of SAGs (lake water SAGs in blue, sedimentary SAGs in red, and SAGs from other taxonomic groups in green) and closely related GTDB genomes (taxa in black). Branch support (see color bar in the upper left) was assessed using SH values, ranging from blue (weak) to green (moderate) to red (strong). The value next to the branch of the common ancestor of the SAGs represents the genealogical

sorting index, ranging from 0 (weakest) to 1 (strongest), which quantifies the strength of SAGs' monophyly. The value in parentheses denotes the P-value of the GSI. In the right panel, the upper graph shows the top 10 metabolic pathways enriched by SLM SAGs, while the lower graph shows the top 10 metabolic pathways enriched by sister taxa. Source data are provided as a Source Data file.

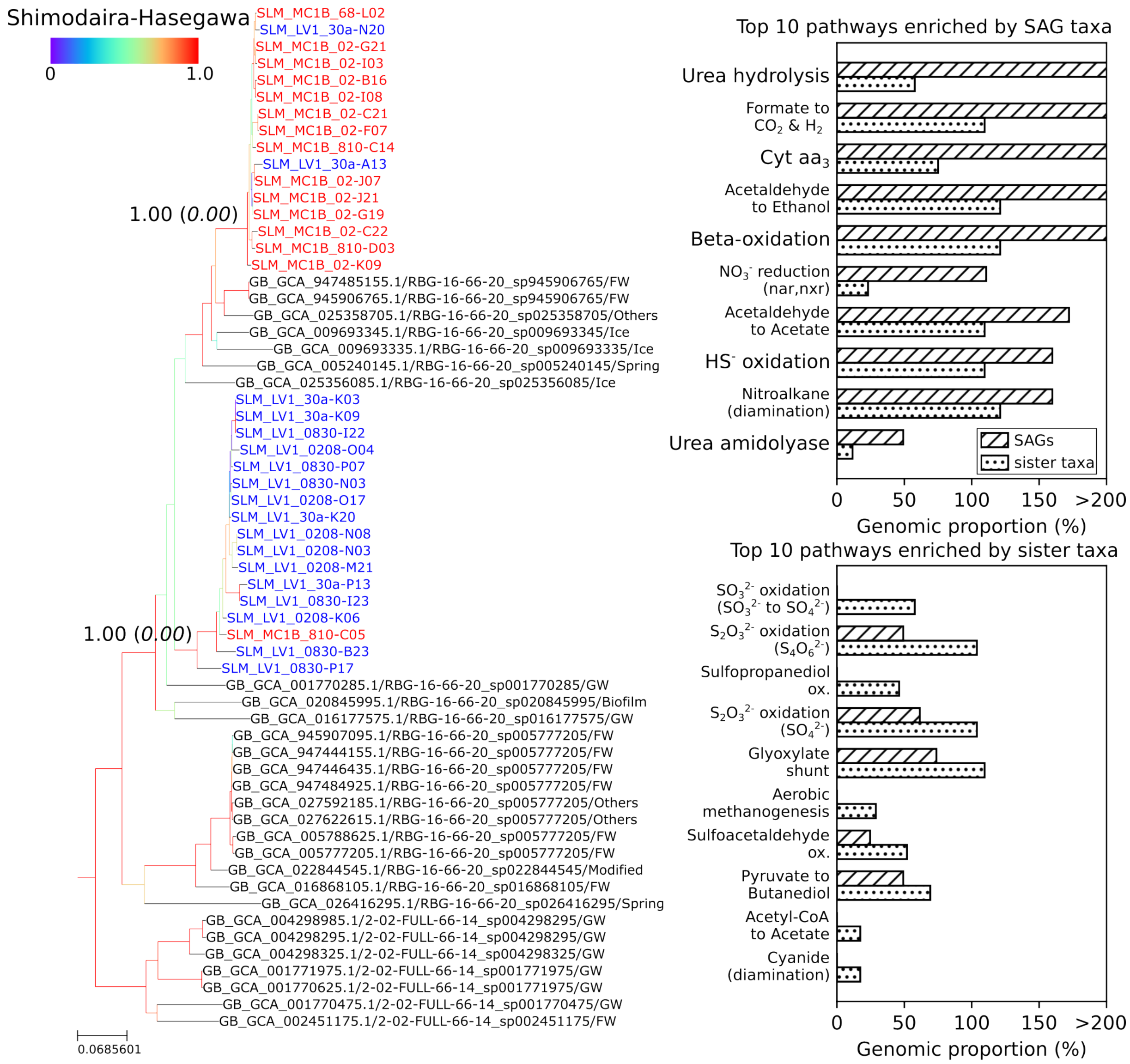


**Supplementary Fig. 17. Phylogenomic trees of the genus RBG-16-66-20.** Maximum-likelihood trees were reconstructed using the GTDB marker gene alignments of SAGs (lake water SAGs in blue, sedimentary SAGs in red, and SAGs from other taxonomic groups in green) and closely related GTDB genomes (taxa in black). Branch support (see color bar in the upper left) was assessed using SH values, ranging from blue (weak) to green (moderate) to red (strong). The value next to the branch of the common ancestor of the SAGs represents the genealogical sorting index, ranging from 0 (weakest) to 1 (strongest), which quantifies the strength of SAGs' monophyly. The value in parentheses denotes the P-value of the GSI. In the right panel, the upper graph shows the top 10 metabolic pathways enriched by SLM SAGs, while the lower graph shows the top 10 metabolic pathways enriched by sister taxa. Source data are provided as a Source Data file.

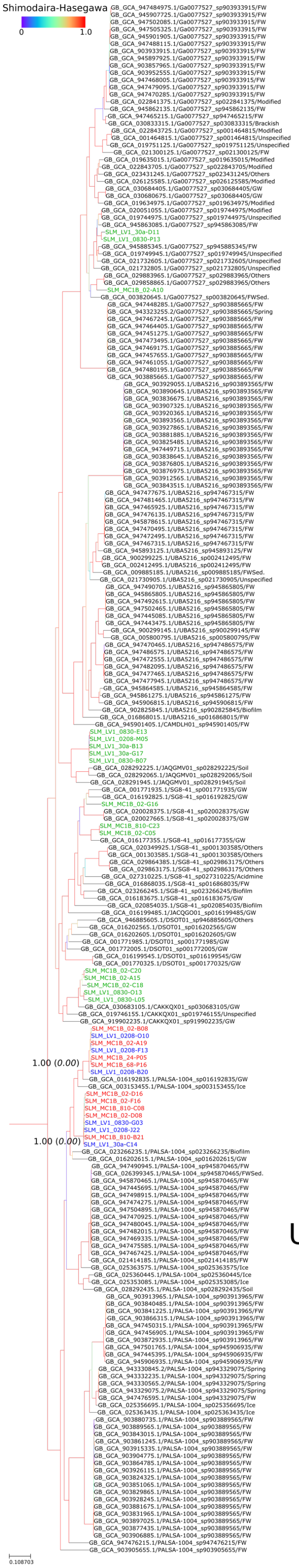

Shimodaira-Hasegawa
0
1.0
1.00 (0.00)
1.00 (0.00)


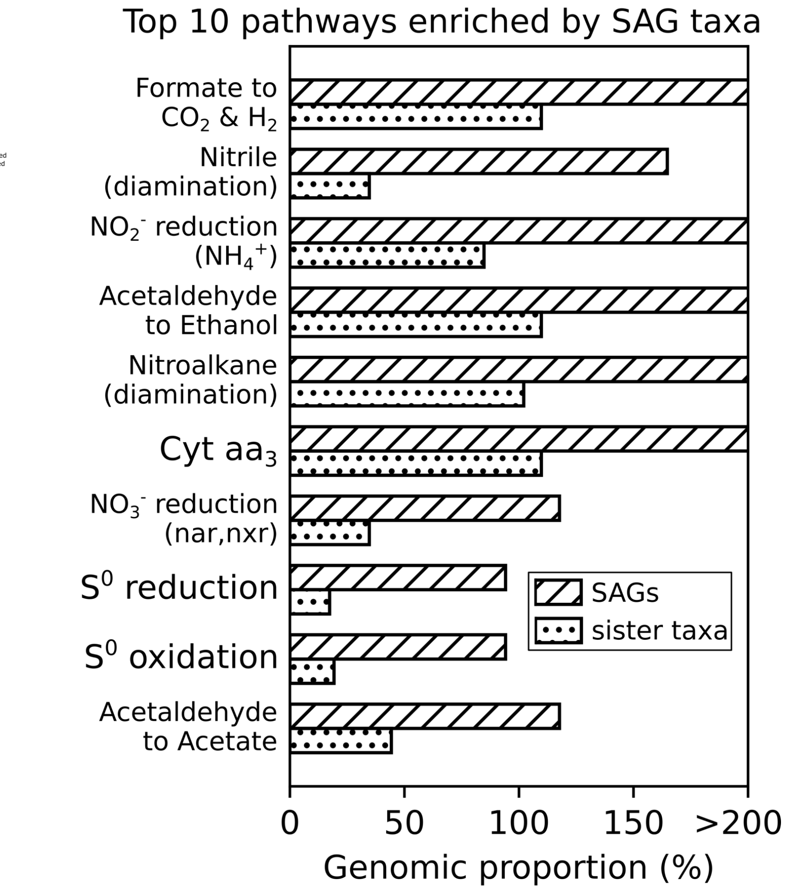

Top 10 pathways enriched by SAG taxa
Formate to $CO_2$ & $H_2$
Nitrile (diamination)
$NO_2^-$ reduction ($NH_4^+$)
Acetaldehyde to Ethanol
Nitroalkane (diamination)
Cyt $aa_3$
$NO_3^-$ reduction (nar,nxr)
$S^0$ reduction
$S^0$ oxidation
Acetaldehyde to Acetate
SAGs
sister taxa
0
50
100
150
>200
Genomic proportion (%)


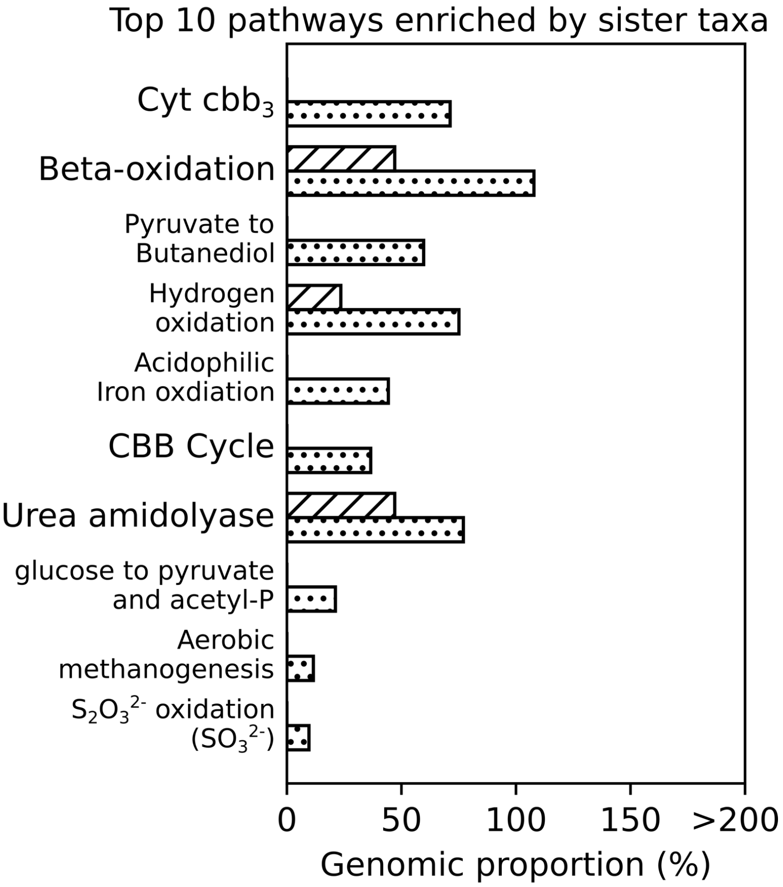

Top 10 pathways enriched by sister taxa
Cyt $cbb_3$
Beta-oxidation
Pyruvate to Butanediol
Hydrogen oxidation
Acidophilic Iron oxdiation
CBB Cycle
Urea amidolyase
glucose to pyruvate and acetyl-P
Aerobic methanogenesis
$S_2O_3^{2-}$ oxidation ($SO_3^{2-}$)
0
50
100
150
>200
Genomic proportion (%)

**Supplementary Fig. 18. Phylogenomic trees of the genus PALSA-1004.** Maximum-likelihood trees were reconstructed using the GTDB marker gene alignments of SAGs (lake water SAGs in blue, sedimentary SAGs in red, and SAGs from other taxonomic groups in green) and closely related GTDB genomes (taxa in black). Branch support (see color bar in the upper left) was assessed using SH values, ranging from blue (weak) to green (moderate) to red (strong). The value next to the branch of the common ancestor of the SAGs represents the genealogical sorting index, ranging from 0 (weakest) to 1 (strongest), which quantifies the strength of SAGs' monophyly. The value in parentheses denotes the P-value of the GSI. In the right panel, the upper graph shows the top 10 metabolic pathways enriched by SLM SAGs, while the lower graph shows the top 10 metabolic pathways enriched by sister taxa. Source data are provided as a Source Data file.

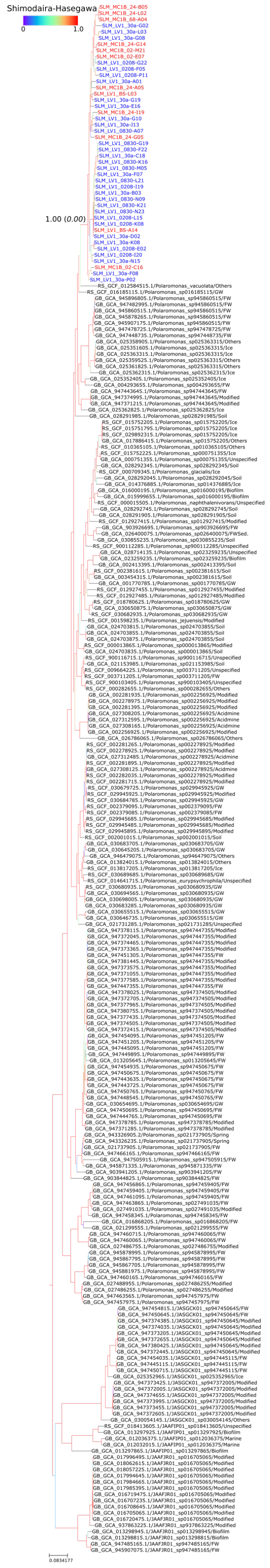
Shimodaira-Hasegawa
0
1.0
1.00 (0.00)

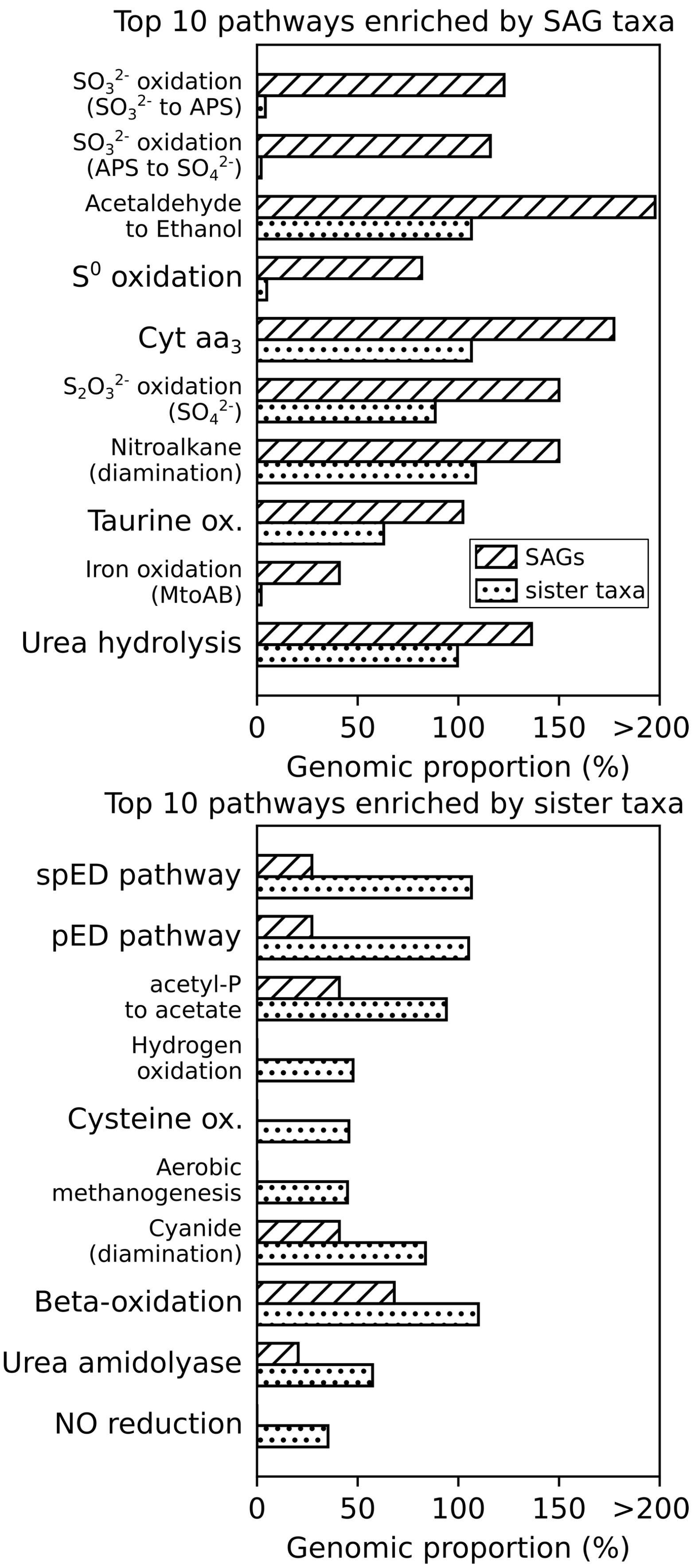
Top 10 pathways enriched by SAG taxa
$SO_3^{2-}$ oxidation ($SO_3^{2-}$ to APS)
$SO_3^{2-}$ oxidation (APS to $SO_4^{2-}$)
Acetaldehyde to Ethanol
$S^0$ oxidation
Cyt $aa_3$
$S_2O_3^{2-}$ oxidation ($SO_4^{2-}$)
Nitroalkane (diamination)
Taurine ox.
Iron oxidation (MtoAB)
Urea hydrolysis
SAGs
sister taxa
0
50
100
150
>200
Genomic proportion (%)
Top 10 pathways enriched by sister taxa
spED pathway
pED pathway
acetyl-P to acetate
Hydrogen oxidation
Cysteine ox.
Aerobic methanogenesis
Cyanide (diamination)
Beta-oxidation
Urea amidolyase
NO reduction
0
50
100
150
>200
Genomic proportion (%)

**Supplementary Fig. 19. Phylogenomic trees of the genus *Polaromonas*.** Maximum-likelihood trees were reconstructed using the GTDB marker gene alignments of SAGs (lake water SAGs in blue, sedimentary SAGs in red, and SAGs from other taxonomic groups in green) and closely related GTDB genomes (taxa in black). Branch support (see color bar in the upper left) was assessed using SH values, ranging from blue (weak) to green (moderate) to red (strong). The value next to the branch of the common ancestor of the SAGs represents the genealogical sorting index, ranging from 0 (weakest) to 1 (strongest), which quantifies the strength of SAGs' monophyly. The value in parentheses denotes the P-value of the GSI. In the right panel, the upper graph shows the top 10 metabolic pathways enriched by SLM SAGs, while the lower graph shows the top 10 metabolic pathways enriched by sister taxa. Source data are provided as a Source Data file.

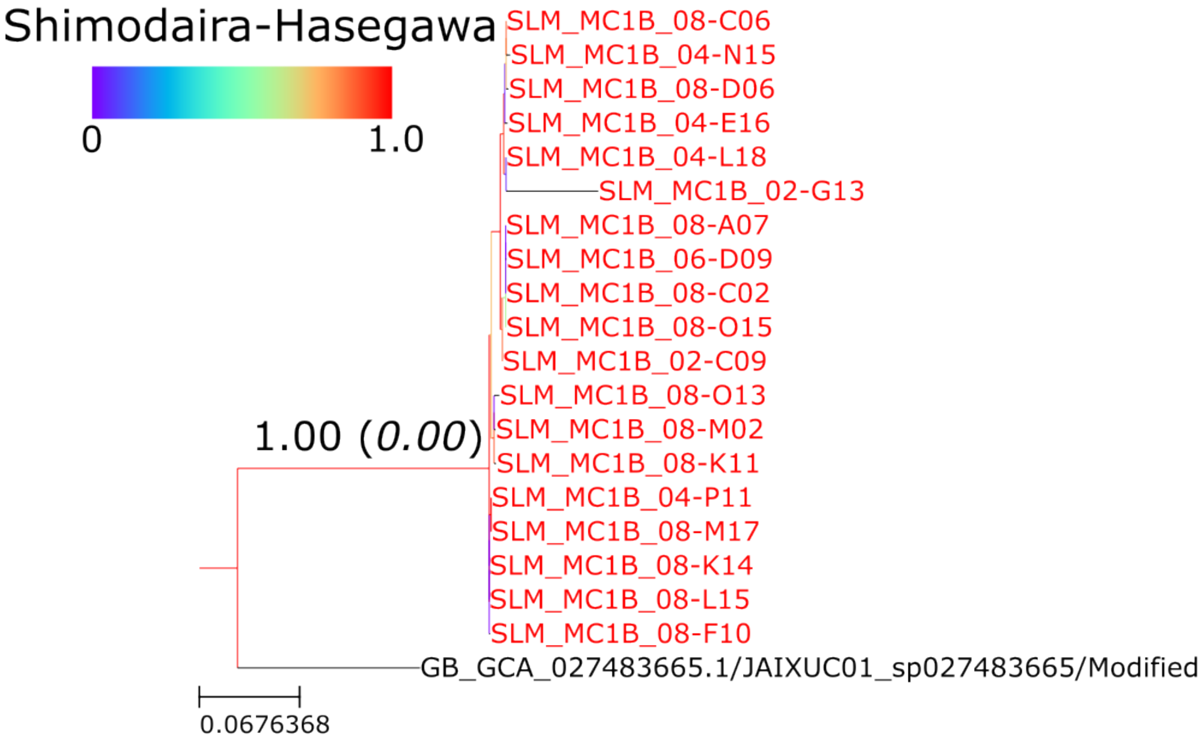


**Supplementary Fig. 20. Phylogenomic trees of Burkholderiaceae incertae sedis.** Maximum-likelihood trees were reconstructed using the GTDB marker gene alignments of SAGs (lake water SAGs in blue, sedimentary SAGs in red, and SAGs from other taxonomic groups in green) and closely related GTDB genomes (taxa in black). Branch support (see color bar in the upper left) was assessed using SH values, ranging from blue (weak) to green (moderate) to red (strong). The value next to the branch of the common ancestor of the SAGs represents the genealogical sorting index, ranging from 0 (weakest) to 1 (strongest), which quantifies the strength of SAGs' monophyly. The value in parentheses denotes the P-value of the GSI. Note that the enrichment of metabolic pathways was omitted since sister taxa were not sufficiently available for this genus. Source data are provided as a Source Data file.

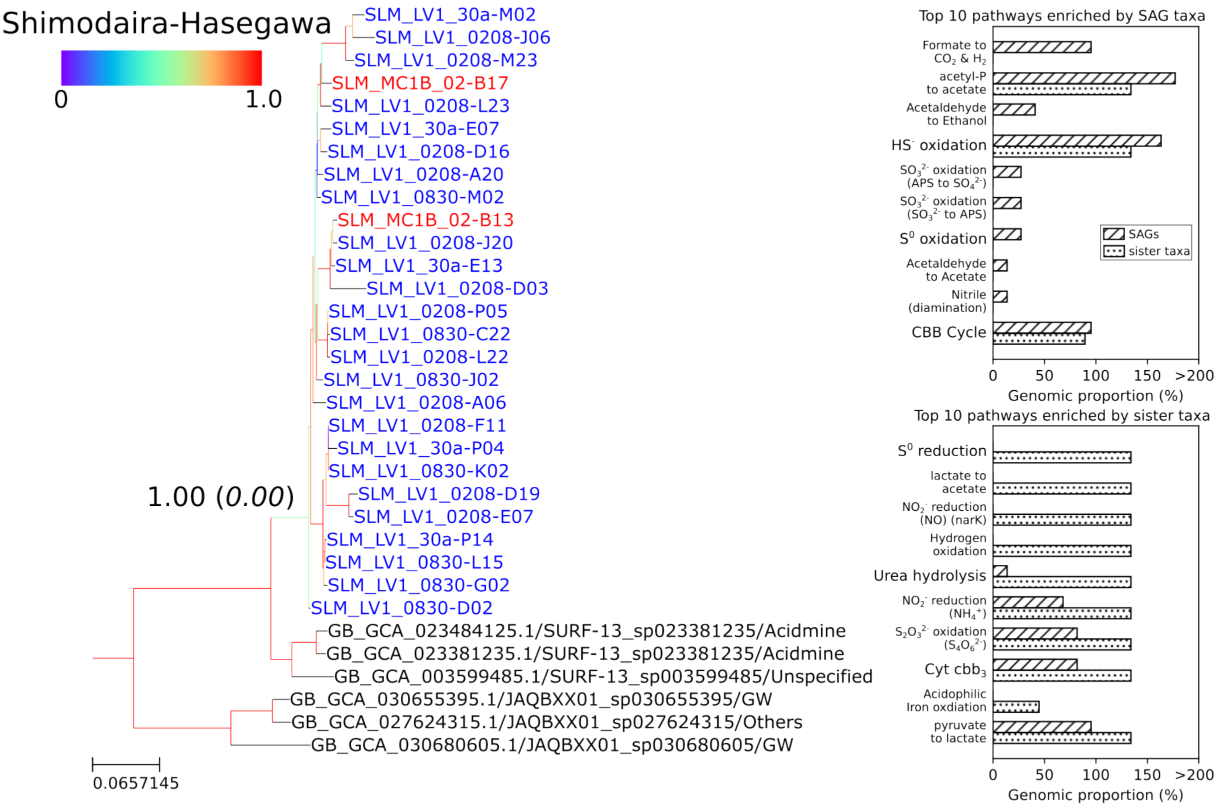


**Supplementary Fig. 21. Phylogenomic trees of the genus SURF-13.** Maximum-likelihood trees were reconstructed using the GTDB marker gene alignments of SAGs (lake water SAGs in blue, sedimentary SAGs in red, and SAGs from other taxonomic groups in green) and closely related GTDB genomes (taxa in black). Branch support (see color bar in the upper left) was assessed using SH values, ranging from blue (weak) to green (moderate) to red (strong). The value next to the branch of the common ancestor of the SAGs represents the genealogical sorting index, ranging from 0 (weakest) to 1 (strongest), which quantifies the strength of SAGs' monophyly. The value in parentheses denotes the P-value of the GSI. In the right panel, the upper graph shows the top 10 metabolic pathways enriched by SLM SAGs, while the lower graph shows the top 10 metabolic pathways enriched by sister taxa. Source data are provided as a Source Data file.

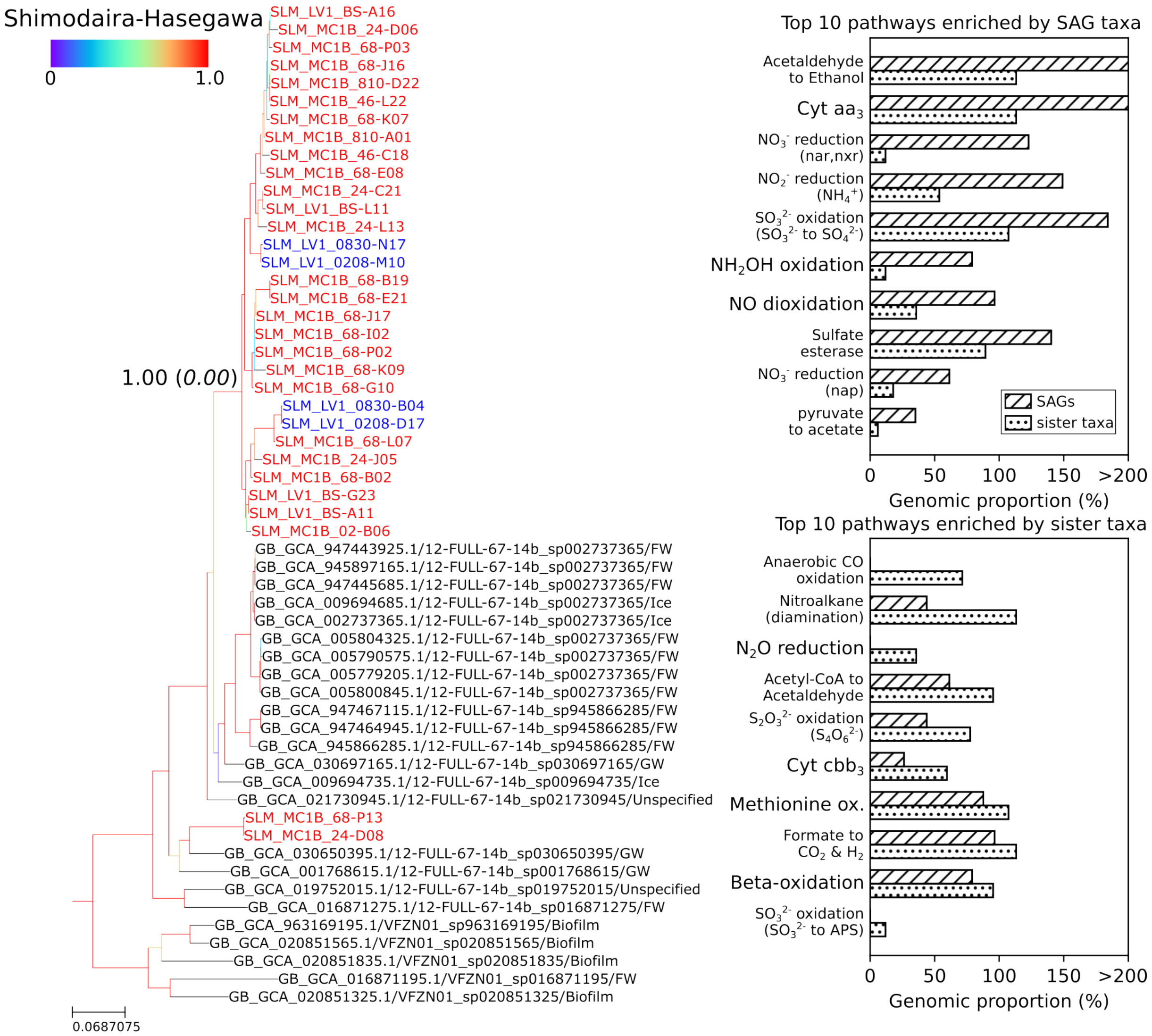


**Supplementary Fig. 22. Phylogenomic trees of the genus 12-FULL-67-14b.** Maximum-likelihood trees were reconstructed using the GTDB marker gene alignments of SAGs (lake water SAGs in blue, sedimentary SAGs in red, and SAGs from other taxonomic groups in green) and closely related GTDB genomes (taxa in black). Branch support (see color bar in the upper left) was assessed using SH values, ranging from blue (weak) to green (moderate) to red (strong). The value next to the branch of the common ancestor of the SAGs represents the genealogical sorting index, ranging from 0 (weakest) to 1 (strongest), which quantifies the strength of SAGs' monophyly. The value in parentheses denotes the P-value of the GSI. In the right panel, the upper graph shows the top 10 metabolic pathways enriched by SLM SAGs, while the lower graph shows the top 10 metabolic pathways enriched by sister taxa. Source data are provided as a Source Data file.

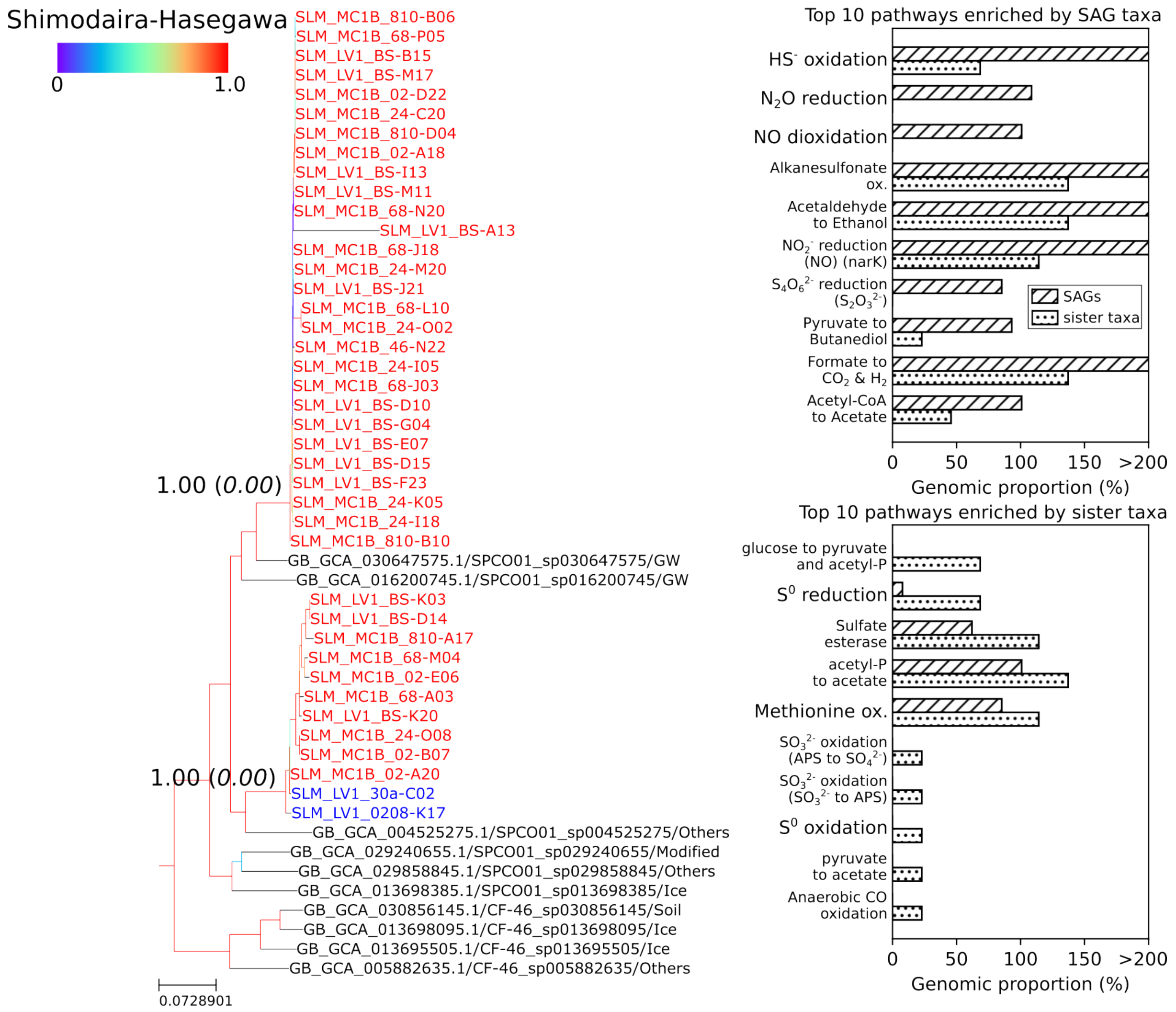


**Supplementary Fig. 23. Phylogenomic trees of the genus SPCO01.** Maximum-likelihood trees were reconstructed using the GTDB marker gene alignments of SAGs (lake water SAGs in blue, sedimentary SAGs in red, and SAGs from other taxonomic groups in green) and closely related GTDB genomes (taxa in black). Branch support (see color bar in the upper left) was assessed using SH values, ranging from blue (weak) to green (moderate) to red (strong). The value next to the branch of the common ancestor of the SAGs represents the genealogical sorting index, ranging from 0 (weakest) to 1 (strongest), which quantifies the strength of SAGs' monophyly. The value in parentheses denotes the P-value of the GSI. In the right panel, the upper graph shows the top 10 metabolic pathways enriched by SLM SAGs, while the lower graph shows the top 10 metabolic pathways enriched by sister taxa. Source data are provided as a Source Data file.

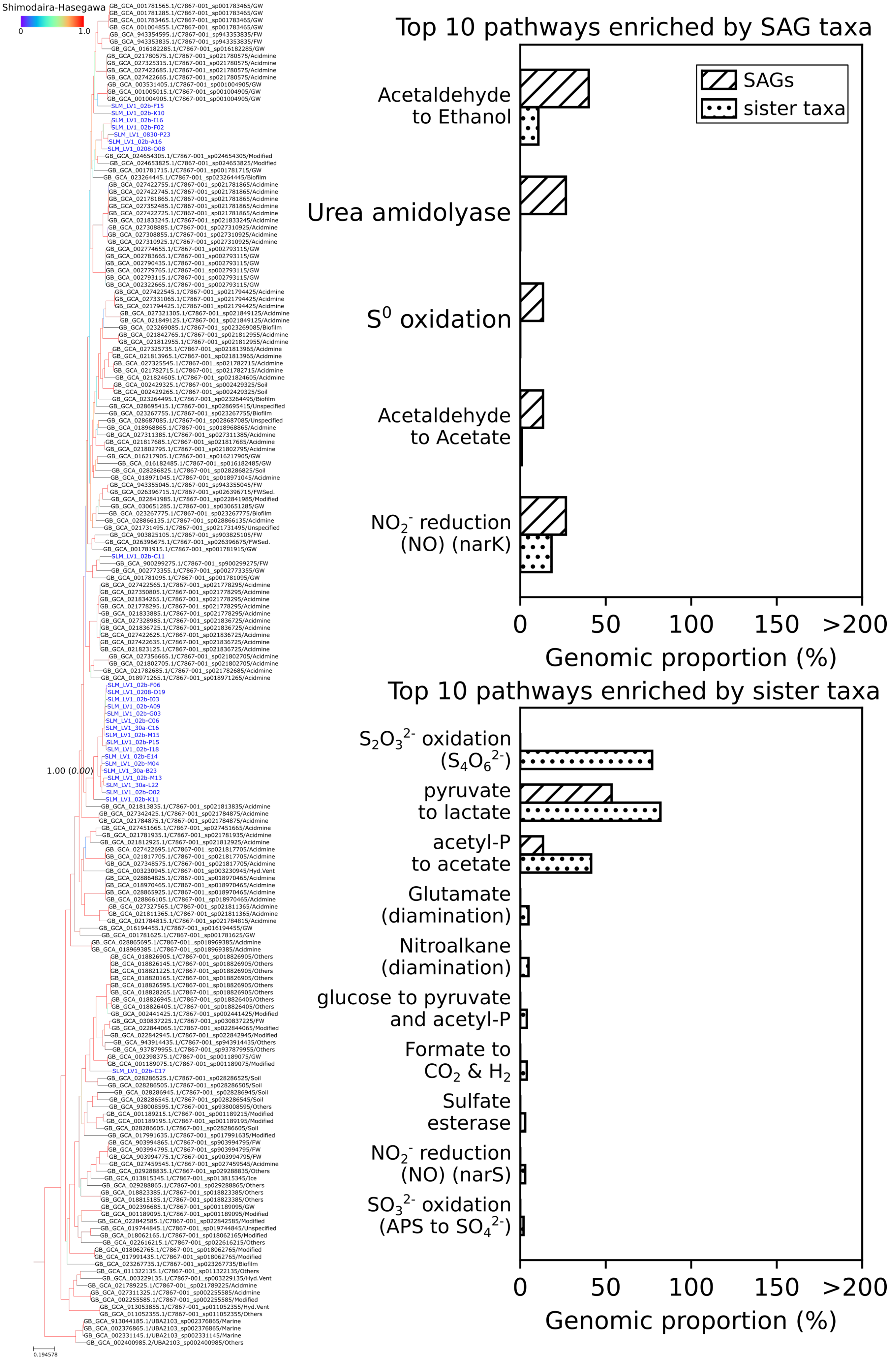

Shimodaira-Hasegawa
0
1.0
1.00 (0.00)
Top 10 pathways enriched by SAG taxa
SAGs
sister taxa
Acetaldehyde to Ethanol
Urea amidolyase
$S^0$ oxidation
Acetaldehyde to Acetate
$NO_2^-$ reduction (NO) (narK)
0
50
100
150
>200
Genomic proportion (%)
Top 10 pathways enriched by sister taxa
$S_2O_3^{2-}$ oxidation ($S_4O_6^{2-}$)
pyruvate to lactate
acetyl-P to acetate
Glutamate (diamination)
Nitroalkane (diamination)
glucose to pyruvate and acetyl-P
Formate to $CO_2$ & $H_2$
Sulfate esterase
$NO_2^-$ reduction (NO) (narS)
$SO_3^{2-}$ oxidation (APS to $SO_4^{2-}$)
0
50
100
150
>200
Genomic proportion (%)

**Supplementary Fig. 24. Phylogenomic trees of the genus C7867-001.** Maximum-likelihood trees were reconstructed using the GTDB marker gene alignments of SAGs (lake water SAGs in blue, sedimentary SAGs in red, and SAGs from other taxonomic groups in green) and closely related GTDB genomes (taxa in black). Branch support (see color bar in the upper left) was assessed using SH values, ranging from blue (weak) to green (moderate) to red (strong). The value next to the branch of the common ancestor of the SAGs represents the genealogical sorting index, ranging from 0 (weakest) to 1 (strongest), which quantifies the strength of SAGs' monophyly. The value in parentheses denotes the P-value of the GSI. In the right panel, the upper graph shows the top 10 metabolic pathways enriched by SLM SAGs, while the lower graph shows the top 10 metabolic pathways enriched by sister taxa. Source data are provided as a Source Data file.

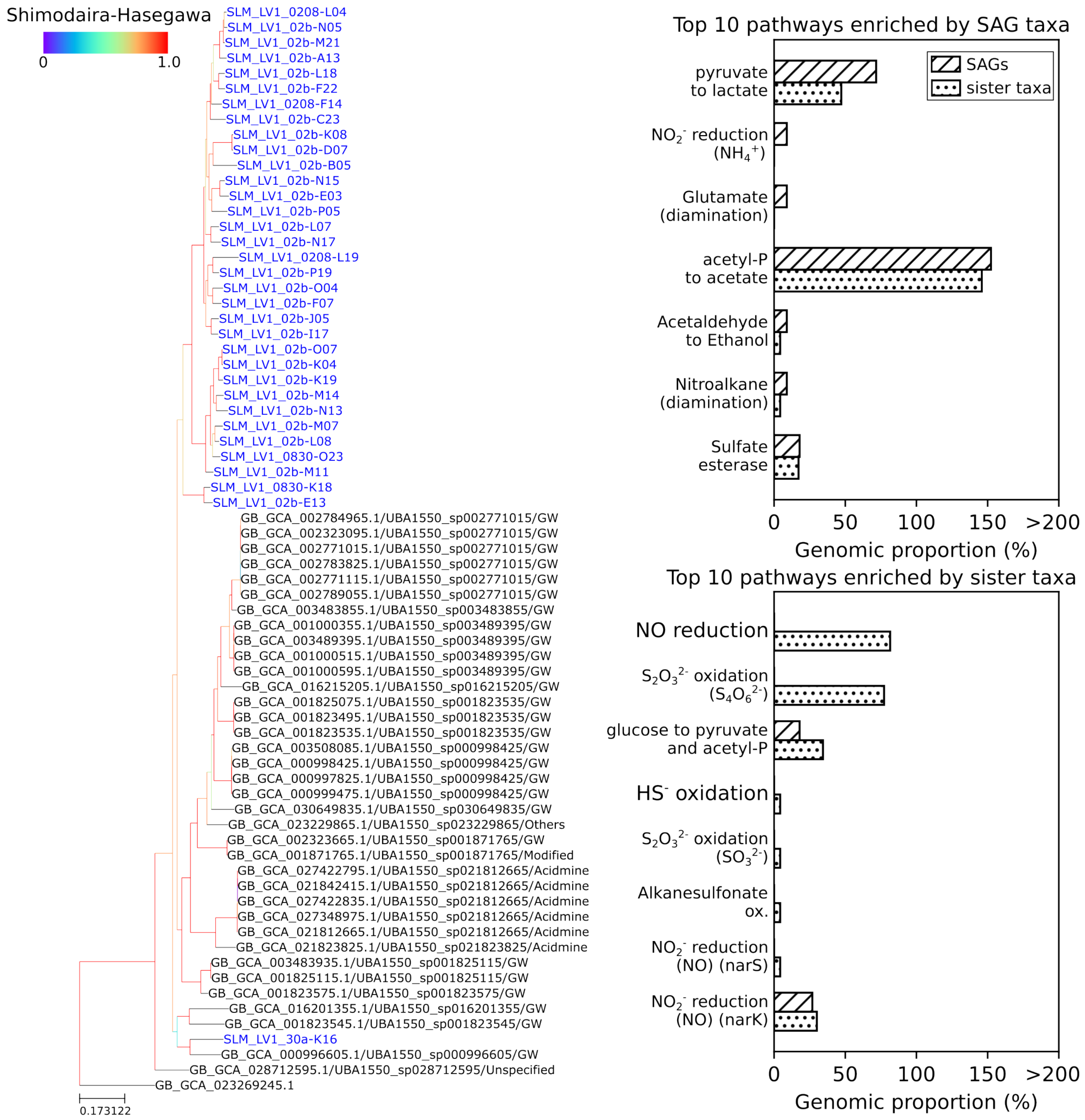


**Supplementary Fig. 25. Phylogenomic trees of the genus UBA1550.** Maximum-likelihood trees were reconstructed using the GTDB marker gene alignments of SAGs (lake water SAGs in blue, sedimentary SAGs in red, and SAGs from other taxonomic groups in green) and closely related GTDB genomes (taxa in black). Branch support (see color bar in the upper left) was assessed using SH values, ranging from blue (weak) to green (moderate) to red (strong). The value next to the branch of the common ancestor of the SAGs represents the genealogical sorting index, ranging from 0 (weakest) to 1 (strongest), which quantifies the strength of SAGs' monophyly. The value in parentheses denotes the P-value of the GSI. In the right panel, the upper graph shows the top 10 metabolic pathways enriched by SLM SAGs, while the lower

graph shows the top 10 metabolic pathways enriched by sister taxa. Source data are provided as a Source Data file.

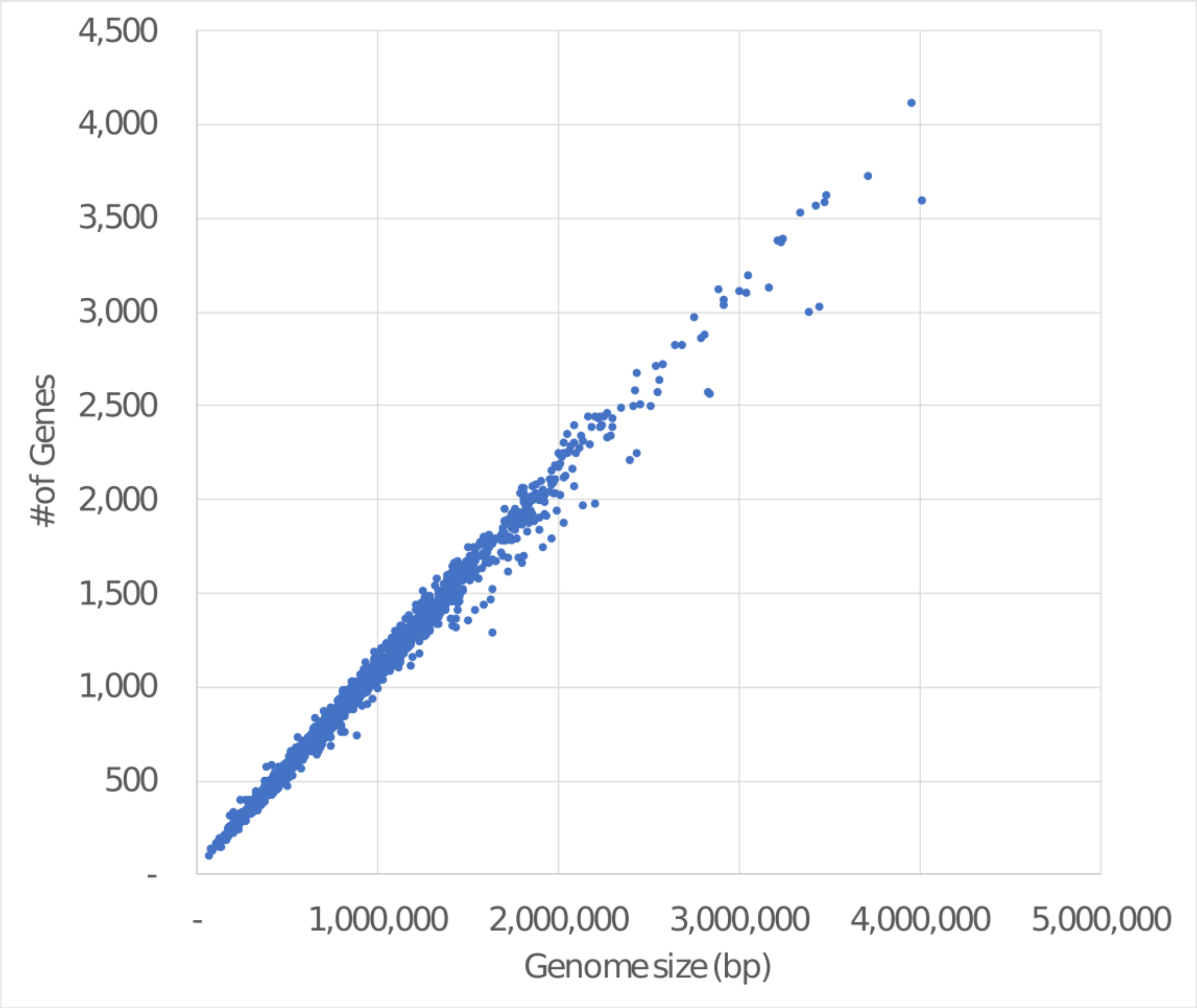


**Supplementary Fig. 26. Correlation between genome size and the number of genes for the 1,374 SAGs.**

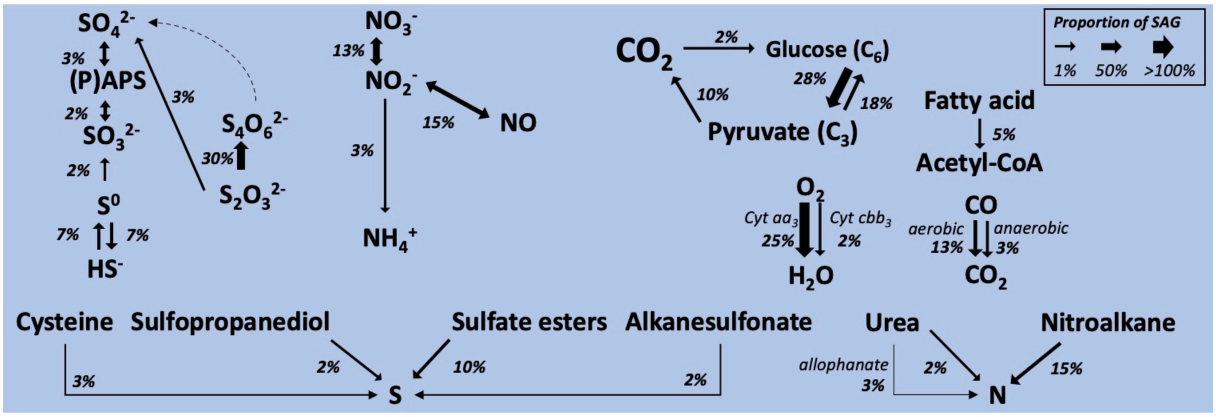


**Supplementary Fig. 27. Metabolic potential inferred from SLM's lake_small_SAGs.** The proportion of 190 lake_small_SAGs involved in a metabolic reaction were calculated regarding incomplete genome recovery as described previously[16] and correlates with the thickness of arrow bodies.

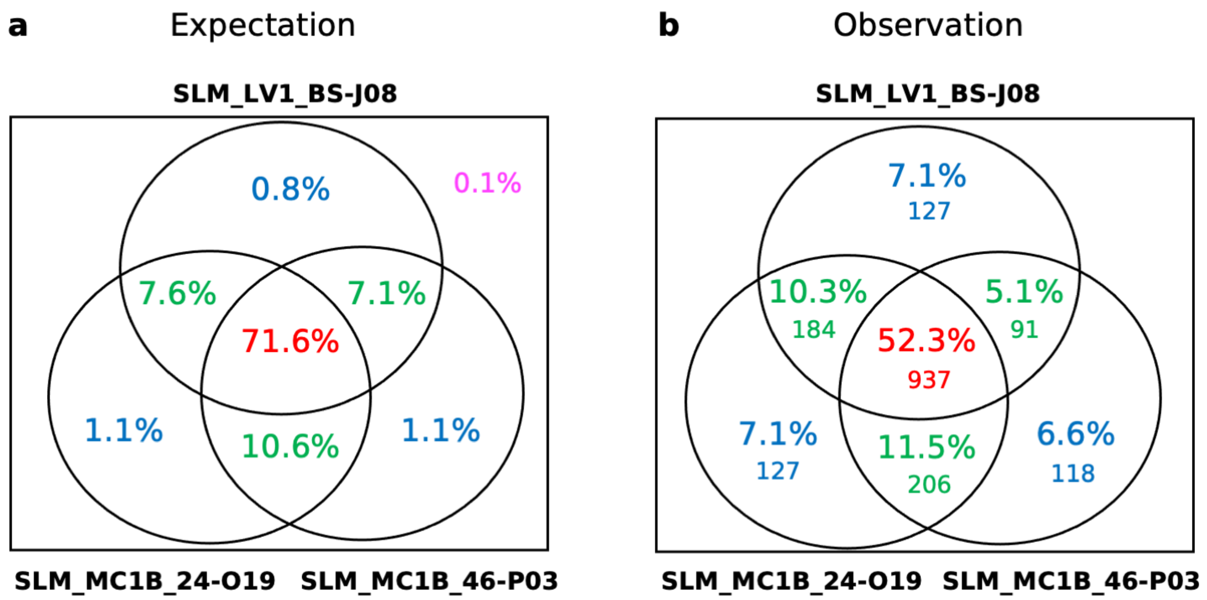


**Supplementary Fig. 28. Genetic heterogeneity between SAGs of the same species.** The proportion of KEGG orthologs shared by highly similar three SAGs (SLM_MC1B_46-P03, SLM_MC1B_24-O19, and SLM_LV1_BS-J08) belonging to the same species were calculated. (**a**) The expected proportion of KEGG orthologs shared by the three incomplete genomes under a null hypothesis that these genomes are completely same in gene content when they are complete genomes. (**b**) The observed proportion of KEGG orthologs shared by the three incomplete genomes. Source data are provided as a Source Data file.

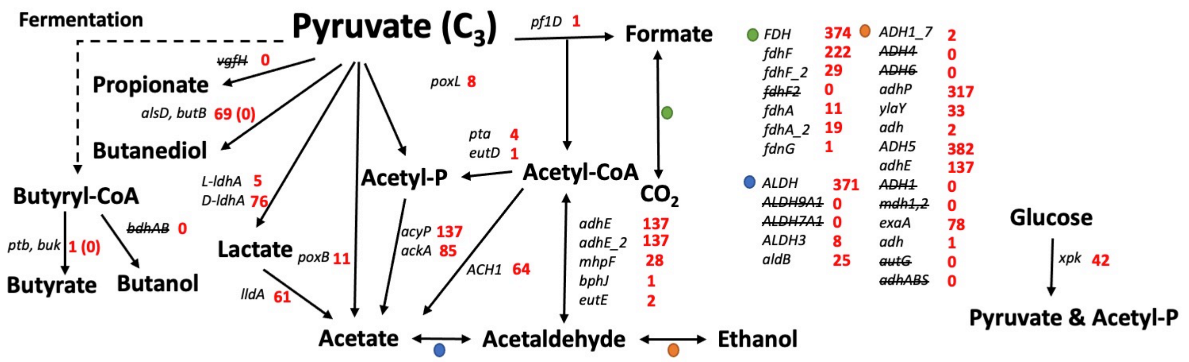


**Supplementary Fig. 29. Fermentation potential exhibited by SLM's SAGs.** The word and number beside an arrow indicate a gene name and the number of SAGs possessing it. Two enzyme pairs (*alsD-butB* and *ptb-buk*) catalyze sequential reactions for butanediol and butyrate, respectively, but the two individual genes for each pair do not co-occur in any SAG.

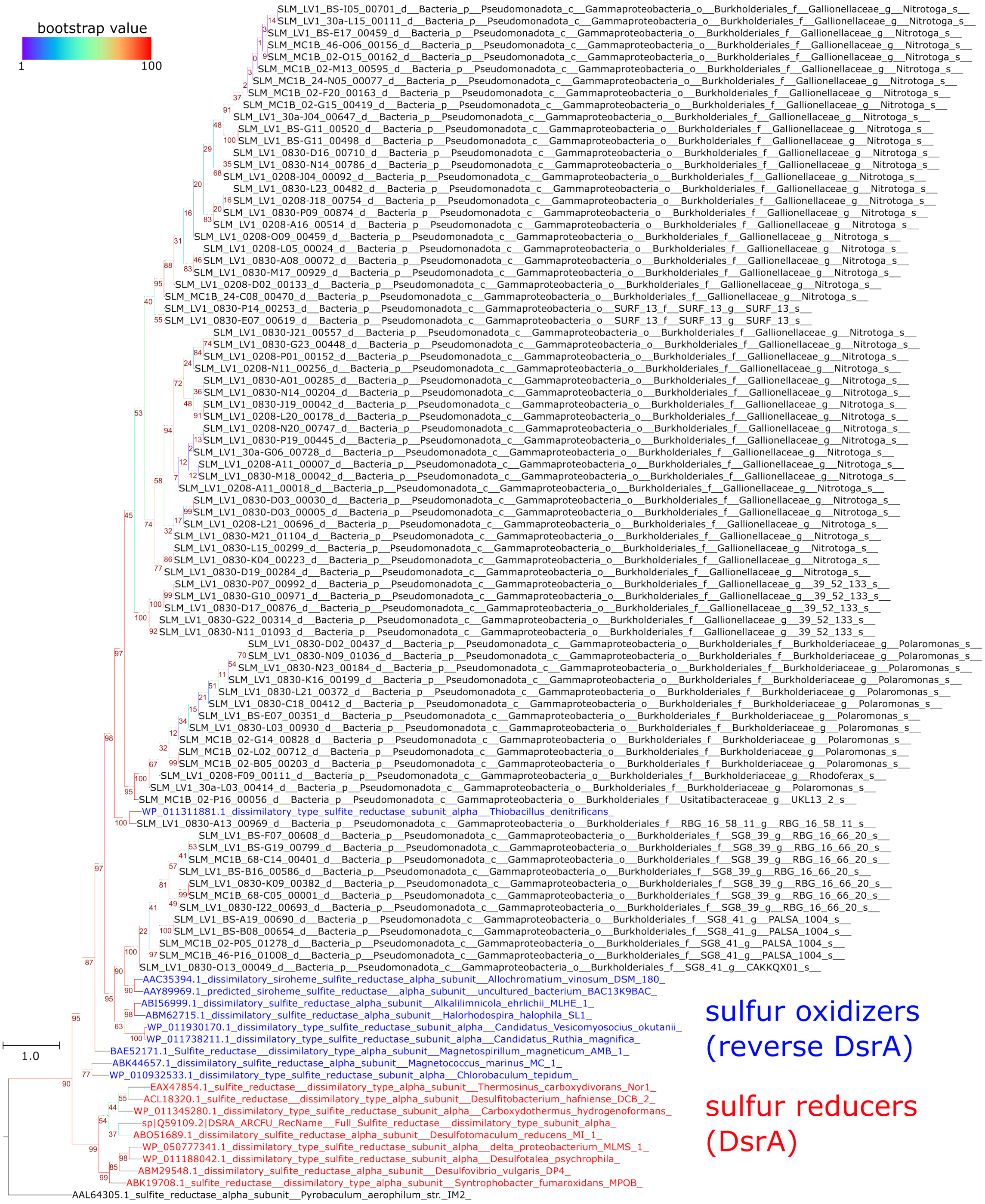


**Supplementary Fig. 30. Phylogenetic tree of the DsrA sequences.** In order to determine whether DsrA proteins from SAGs are responsible for sulfur oxidation or sulfite reduction, a maximum-likelihood tree was reconstructed using RAxML with sequences of SAGs (taxa in black) and experimentally proved oxidizers (taxa in blue) and reducers (taxa in red)[17]. Branch support (see color bar in the upper left) was assessed using bootstrap values, ranging from

blue (weak) to green (moderate) to red (strong). The values next to internal nodes are also bootstrap values. Source data are provided as a Source Data file.

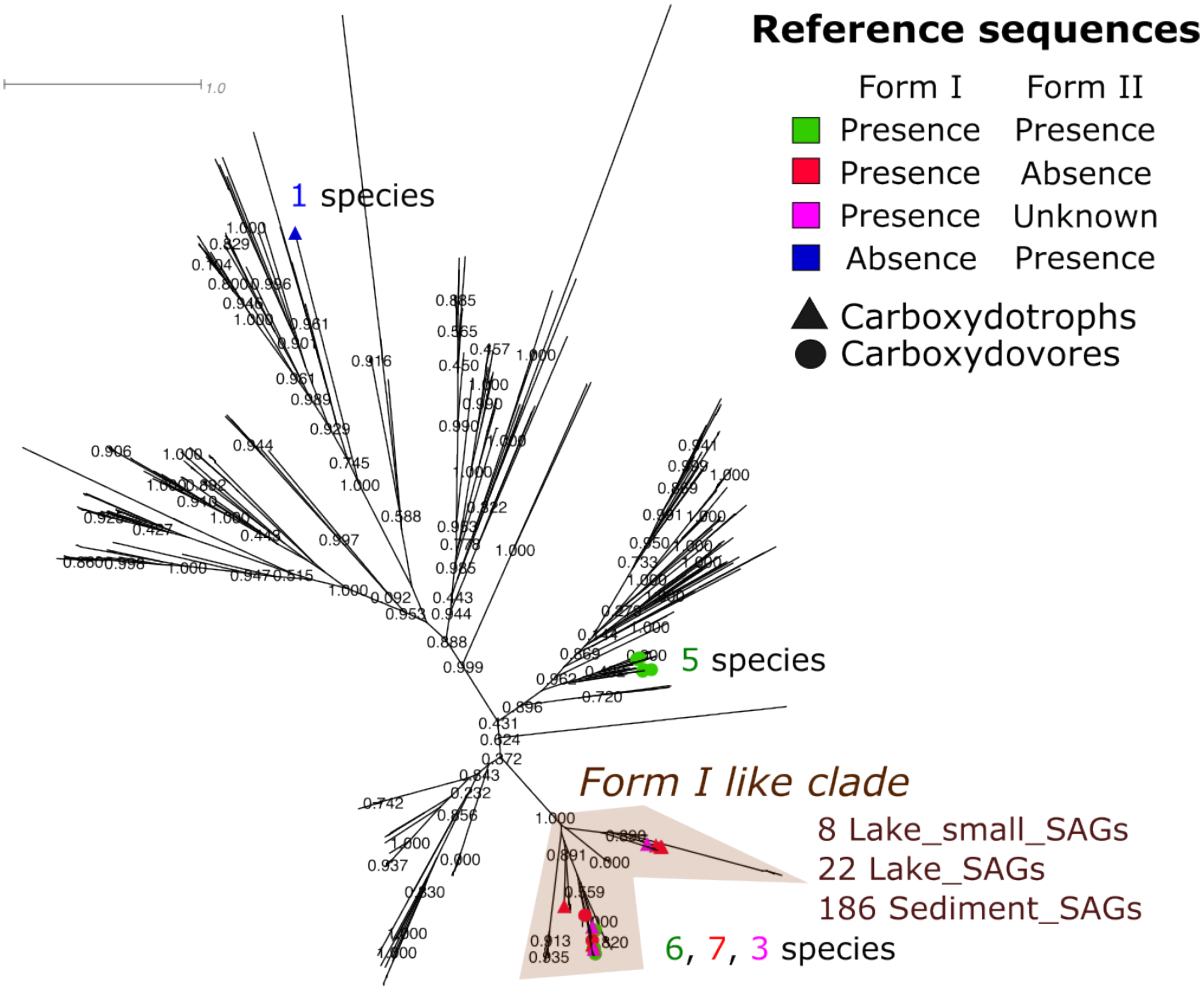


**Supplementary Fig. 31. Phylogenetic tree of CoxL.** In order to separate the Form I CoxL sequences from the Form II CoxL, a maximum-likelihood trees were reconstructed using FastTree v2.1.11[18] with sequences of SAGs and a previous study[19]. Bootstrap confidence values were given at nodes of the tree. Experimentally verified carboxydotrohps and carboxydovores were indicated by triangles and circles at the taxon tips, respectively. The reference CoxL sequences were classified into those originating from genomes possessing both Forms I and II (taxa in green), genomes possessing only Form I (taxa in red), genomes possessing only Form II (taxa in blue) and genomes possessing Form I with uncertain existence of Form II (taxa in magenta). From the ancestral node pointing to the most recent common ancestor that encompasses all of the reference form I CoxL sequences, a derived monophyletic group was considered ‘Form I like clade’. A total of 216 SAGs were included in this clade, where form II reference CoxL sequences were not found. Source data are provided as a Source Data file.

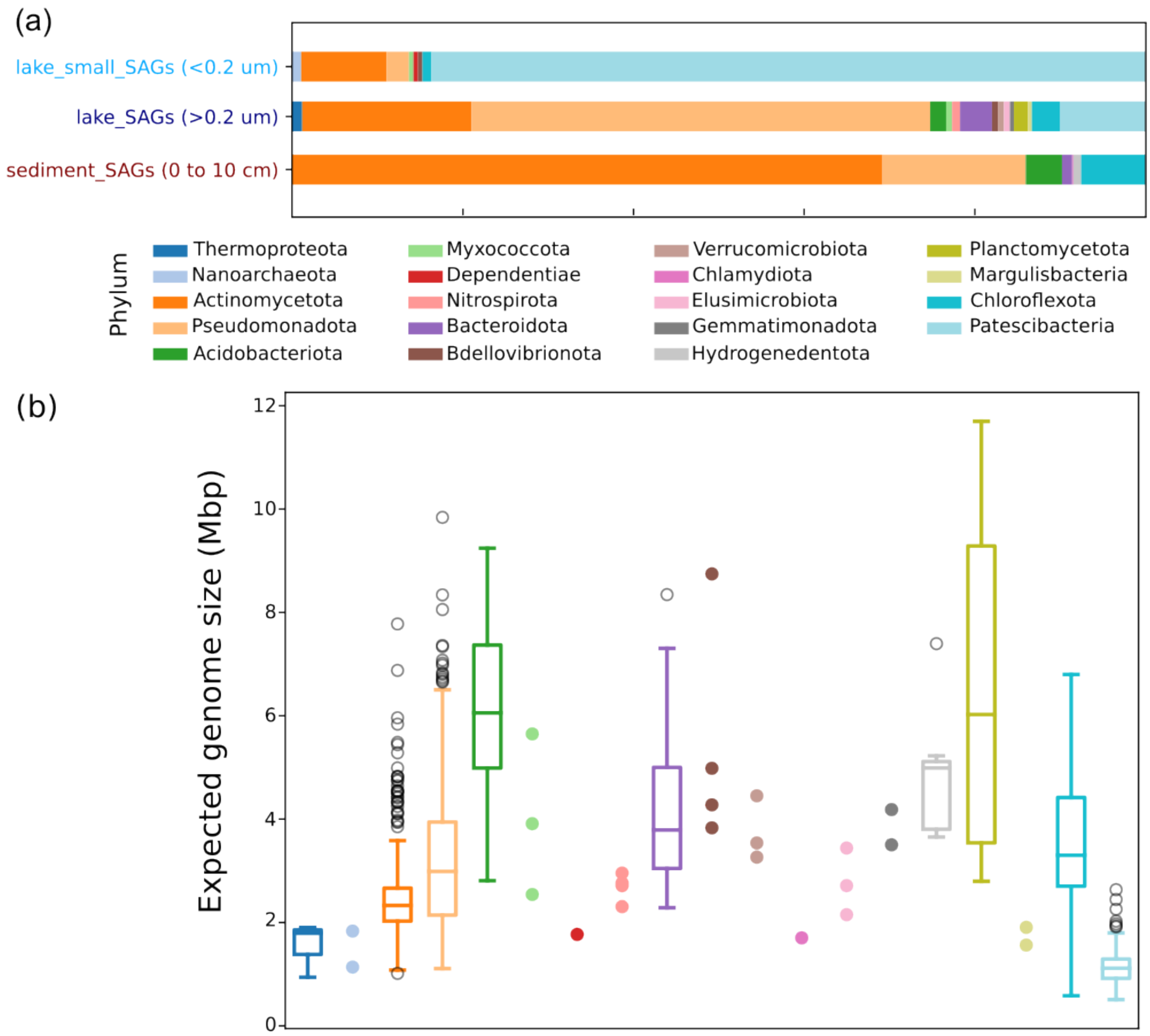


**Supplementary Fig. 32. Taxonomic composition and genome size distribution of 1,374 SAGs from SLM.** (a) Relative abundance of prokaryotic phyla for three sample sets: lake_small_SAGs (190 SAGs), lake_SAGs (428 SAGs), and sediment_SAGs (756 SAGs). The abundance was determined by counting the number of SAGs assigned to each phylum. (b) Boxplot showing genome sizes (Mbp) of 1,374 SAGs grouped by phylum. The expected genome size was estimated by dividing the observed genome size by the CheckM genome completeness value.

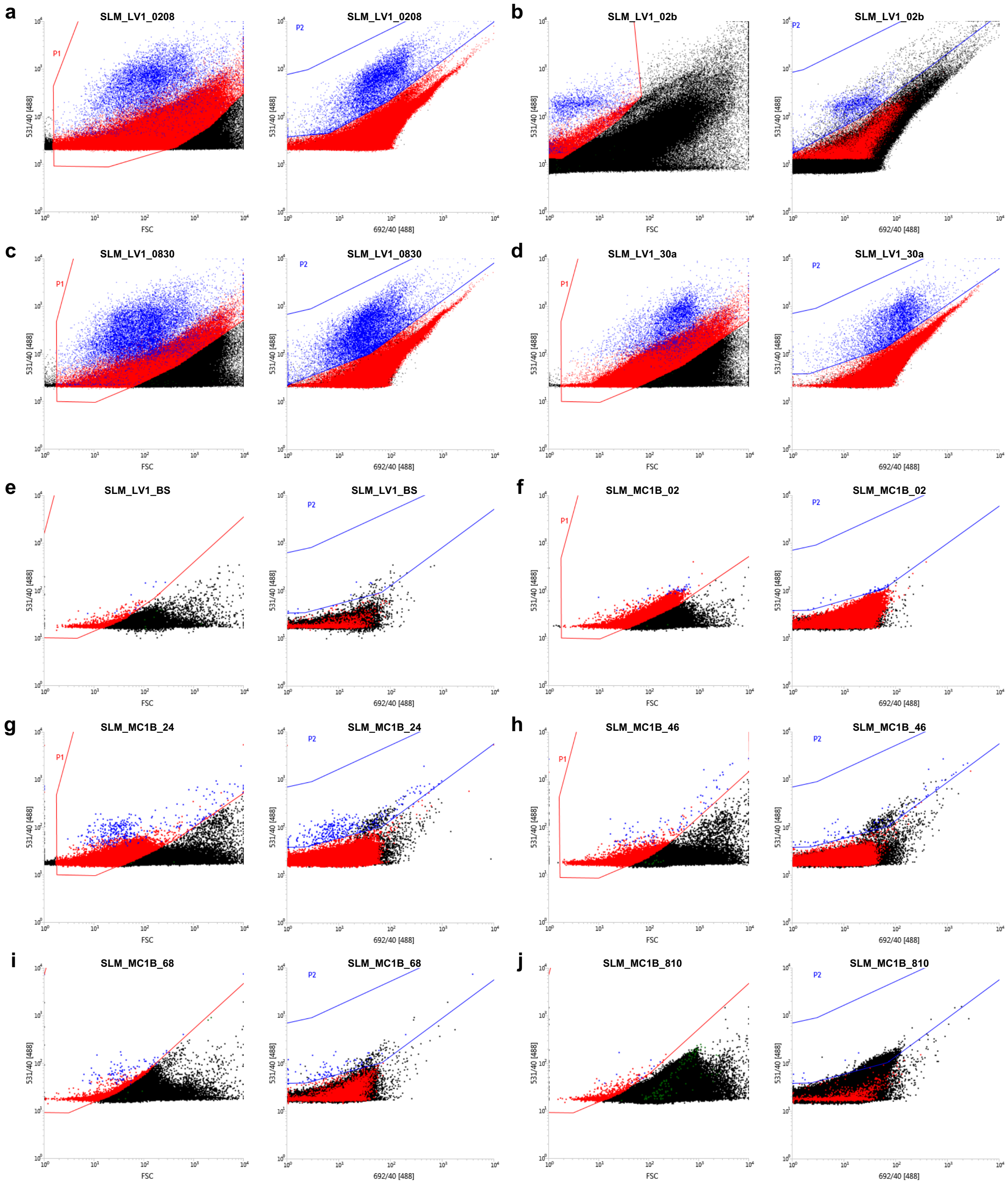


**Supplementary Fig. 33. Flow cytometric characterization and gating strategy for cell sorting of SLM samples.** Flow cytometry profiles are shown for 10 samples (a–j). In each sample, the left panel shows forward scatter (FSC) versus SYTO 9 fluorescence at 531 nm, while the right panel shows autofluorescence at 692 nm versus SYTO 9 fluorescence at 531 nm. Cells were initially gated (P1 gate) based on cell size (FSC) and nucleic acid content (531 nm fluorescence intensity). Subsequently, populations from the P1 gate were further refined

(P2 gate) by selecting cells with relatively high SYTO 9 fluorescence at 531 nm and low autofluorescence at 692 nm. All procedures were performed at the Single Cell Genomics Center (SCGC), Bigelow Laboratory for Ocean Sciences.

## References


1 Priscu, J. C. *et al.* Scientific access into Mercer Subglacial Lake: scientific objectives, drilling operations and initial observations. *Annals of Glaciology* **62**, 340–352 (2021).

2 Davis, C. L. *et al.* Biogeochemical and historical drivers of microbial community composition and structure in sediments from Mercer Subglacial Lake, West Antarctica. *ISME communications* **3**, 8 (2023).

3 Davis, C. L. *Ecology of Subglacial Lake Microbial Communities in West Antarctica*. (University of Florida, 2022).

4 Stepanauskas, R. *et al.* Improved genome recovery and integrated cell-size analyses of individual uncultured microbial cells and viral particles. *Nat Commun* **8**, 84 (2017). https://doi.org/10.1038/s41467-017-00128-z

5 Bankevich, A. *et al.* SPAdes: a new genome assembly algorithm and its applications to single-cell sequencing. *Journal of computational biology* **19**, 455–477 (2012).

6 Parks, D. H., Imelfort, M., Skennerton, C. T., Hugenholtz, P. & Tyson, G. W. CheckM: assessing the quality of microbial genomes recovered from isolates, single cells, and metagenomes. *Genome research* **25**, 1043–1055 (2015).

7 Astashyn, A. *et al.* Rapid and sensitive detection of genome contamination at scale with FCS-GX. *bioRxiv* (2023). https://doi.org/10.1101/2023.06.02.543519

8 Pruesse, E., Peplies, J. & Glöckner, F. O. SINA: accurate high-throughput multiple sequence alignment of ribosomal RNA genes. *Bioinformatics* **28**, 1823–1829 (2012).

9 Stamatakis, A. RAxML version 8: a tool for phylogenetic analysis and post-analysis of large phylogenies. *Bioinformatics* **30**, 1312–1313 (2014).

10 Darriba, D. *et al.* ModelTest-NG: a new and scalable tool for the selection of DNA and protein evolutionary models. *Molecular biology and evolution* **37**, 291–294 (2020).

11 Schloter, M., Lebuhn, M., Heulin, T. & Hartmann, A. Ecology and evolution of bacterial microdiversity. *FEMS microbiology reviews* **24**, 647–660 (2000).

12 Vick-Majors, T. J. *et al.* Physiological ecology of microorganisms in subglacial

Lake Whillans. *Frontiers in microbiology* **7**, 1705 (2016).

13 Weller, C. & Wu, M. A generation-time effect on the rate of molecular evolution in bacteria. *Evolution* **69**, 643-652 (2015).

14 Albanese, D. *et al.* Pre-Cambrian roots of novel Antarctic cryptoendolithic bacterial lineages. *Microbiome* **9**, 1-15 (2021).

15 Achberger, A. M. *et al.* Microbial community structure of subglacial lake Whillans, West Antarctica. *Frontiers in microbiology* **7**, 1457 (2016).

16 Acker, M. *et al.* Phosphonate production by marine microbes: exploring new sources and potential function. *Proceedings of the National Academy of Sciences* **119**, e2113386119 (2022).

17 Tully, B. J., Wheat, C. G., Glazer, B. T. & Huber, J. A. A dynamic microbial community with high functional redundancy inhabits the cold, oxic subseafloor aquifer. *The ISME journal* **12**, 1-16 (2018).

18 Price, M. N., Dehal, P. S. & Arkin, A. P. FastTree: computing large minimum evolution trees with profiles instead of a distance matrix. *Molecular biology and evolution* **26**, 1641-1650 (2009).

19 King, G. M. & Weber, C. F. Distribution, diversity and ecology of aerobic CO-oxidizing bacteria. *Nature Reviews Microbiology* **5**, 107-118 (2007).
