# Supplementary material for: Genetic isolation and metabolic complexity of an Antarctic subglacial microbiome
Source: Nat Commun. 2025 Aug 18;16:7501. doi: 10.1038/s41467-025-62753-3 (PMC12361377; doi:10.1038/s41467-025-62753-3)
Supplement: Supplementary file 2 — Description of Additional Supplementary Files [file 41467_2025_62753_MOESM2_ESM.pdf]

Description of Additional Supplementary Files

File Name: Supplementary Data 1
Description: Genome statistics of 1,374 single cell genomes

File Name: Supplementary Data 2
Description: The number of genomes across samples every taxonomic group from domain to species

File Name: Supplementary Data 3
Description: Dictionary for defining the relationships between keywords and habitat flags. Keywords correspond to genomic isolation sources, and habitat flags are broad categories of habitats encompassing keywords. Searching keywords against genomic isiolation information was made in case-insensitive manner, dealing with plural forms.

File Name: Supplementary Data 4
Description: Habitat flag (column) and Habitat (row). See Results and Supplementary Data 5 to understand how a genomic habitat is determined based on Supplementary Data 3 & 4. The similar habitats of "FW", "FWsed", "GW", "Spring" and "Ice" were combined into a freshwater origin in this study.

File Name: Supplementary Data 5
Description: Habitat decision (the last column in pink) for GTDB genomes based on their metadata (Bioproject & Biosample) referring to Habitat flags and keywords (Supplementary Data 3 and 4)

File Name: Supplementary Data 6
Description: Accessions and functions of genes involving single step pathways

File Name: Supplementary Data 7
Description: Accessions and fucntions of genes involving multi-step pathways

File Name: Supplementary Data 8
Description: The presence and absence of metabolic pathways across 1,374 SAGs

File Name: Supplementary Data 9
Description: Statistical difference of metabolic potential betweeen high complete genomes and low complete genomes. Two-sided empirical P values were computed by comparing the observed genomic proportion to 1,000 iterations using artificially reduced genomes. Adjusted P values account for multiple hypothesis testing.

File Name: Supplementary Data 10
Description: Comparision of genomic proportions of 79 metabolic pathways between SLM SAGs and GTDB genomes for the 18 most abundant genera
