# Supplementary material for: Genetic isolation and metabolic complexity of an Antarctic subglacial microbiome
Source: Nat Commun. 2025 Aug 18;16:7501. doi: 10.1038/s41467-025-62753-3 (PMC12361377; doi:10.1038/s41467-025-62753-3)
Supplement: Supplementary file 13 — Reporting summary [file 41467_2025_62753_MOESM13_ESM.pdf]

nature portfolio

Corresponding author(s): Kyung Mo Kim, John Priscu, Ok-Sun Kim

Last updated by author(s): Jul 24, 2025

Reporting Summary

Nature Portfolio wishes to improve the reproducibility of the work that we publish. This form provides structure for consistency and transparency in reporting. For further information on Nature Portfolio policies, see our Editorial Policies and the Editorial Policy Checklist.

Statistics

For all statistical analyses, confirm that the following items are present in the figure legend, table legend, main text, or Methods section.

n/a	Confirmed	
☐	☒	The exact sample size (*n*) for each experimental group/condition, given as a discrete number and unit of measurement
☐	☒	A statement on whether measurements were taken from distinct samples or whether the same sample was measured repeatedly
☐	☒	The statistical test(s) used AND whether they are one- or two-sided<br>*Only common tests should be described solely by name; describe more complex techniques in the Methods section.*
☒	☐	A description of all covariates tested
☐	☒	A description of any assumptions or corrections, such as tests of normality and adjustment for multiple comparisons
☐	☒	A full description of the statistical parameters including central tendency (e.g. means) or other basic estimates (e.g. regression coefficient) AND variation (e.g. standard deviation) or associated estimates of uncertainty (e.g. confidence intervals)
☐	☒	For null hypothesis testing, the test statistic (e.g. *F*, *t*, *r*) with confidence intervals, effect sizes, degrees of freedom and *P* value noted<br>*Give P values as exact values whenever suitable.*
☒	☐	For Bayesian analysis, information on the choice of priors and Markov chain Monte Carlo settings
☒	☐	For hierarchical and complex designs, identification of the appropriate level for tests and full reporting of outcomes
☒	☐	Estimates of effect sizes (e.g. Cohen's *d*, Pearson's *r*), indicating how they were calculated

Our web collection on statistics for biologists contains articles on many of the points above.

Software and code

Policy information about availability of computer code

Data collection	In December 2018, we successfully drilled through 1,087 m of ice and directly accessed Mercer Subglacial Lake, West Antarctica. Both lake water and surficial sediments were sampled using McLane WTS-LV and UWITEC multicorer, respectively. Particles in the water were concentrated and fractionated in-situ into four samples based on different pore diameters (>3 µm, 3 to 0.8 µm, 6 0.8 to 0.2 µm, and <0.2 µm). Five sediment samples were prepared from the top 10 cm of a sediment core that was split every 2 cm. In addition, a bulk surface sediment was collected from a sediment catcher attached to the in-situ water filtration system. Owing to low concentrations of extractable DNA from the samples, we failed to generate microbial genomes using the shotgun metagenomics. We thus applied a single-cell genomic approach that includes FACS, genome amplification and Illumina sequencing, and finally obtained a total of 1,374 single-cell amplified genomes, where 756 genomes were recovered from sediments and 618 from the water column.
Data analysis	The Illumina sequencing raw reads of the 1,374 genomes were quality-filtered using Trimmomatic v0.39, BWA v0.7.17, and kmernorm v1.05. The remaining reads were assembled using SPAdes v3.13.0, and both completeness and contamination of the assemblies were evaluated using CheckM v1.1.3. Taxonomic assignments of the genomes were conducted using GTDB-Tk v2.0.0. Prokka v1.13 yielded both structural, functional annotations. Metabolic potential of the genomes was evaluated using KEGG database release 2020-03-23 and ko2pathway. High-quality genomes were artificially reduced by genome complete reduction pipeline. Phylogenomic trees were reconstructed using RAxML v8.2.12 and FastTree v2.1.11, and visualized using Dendroscope v3.5.10 and ETE v3.1.3. Habitat of public genomes were classified using genome habitat classification pipeline. The genetic distance between genomes was calculated using FastANI. The 16S rRNA amplicon sequences were analyzed using Cutadapt v4.2, DADA2 v1.25.2, VSEARCH v2.24 and vegan package v2.6-4 in R v4.3.1. Network construction based on genomic co-occurrence of ecologically relevant genomes was conducted using CoNet v1.1.1 of Cytoscape v3.9.0 and ClusterViz v1.0.3. The custom Python scripts used in this study are available at the following repositories: 1) habitat classification of microorganisms based on GTDB and NCBI metadata at https://github.com/kyuinHwang/genome-habitat-classification-pipeline, 2) KO-based inference of metabolic potential in microbial genomes at https://github.com/kyuinHwang/ko2pathway, and 3) microbial genome reduction to specified

completeness levels at https://github.com/kyuinHwang/genome-completeness-reduction-pipeline.

For manuscripts utilizing custom algorithms or software that are central to the research but not yet described in published literature, software must be made available to editors and reviewers. We strongly encourage code deposition in a community repository (e.g. GitHub). See the Nature Portfolio guidelines for submitting code & software for further information.

Data

Policy information about availability of data

All manuscripts must include a data availability statement. This statement should provide the following information, where applicable:

- Accession codes, unique identifiers, or web links for publicly available datasets
- A description of any restrictions on data availability
- For clinical datasets or third party data, please ensure that the statement adheres to our policy

The genome sequence data generated in this study have been deposited in NCBI under the project PRJNA1084198 [https://www.ncbi.nlm.nih.gov/bioproject/PRJNA1084198/]. Source data are provided within this paper.

Research involving human participants, their data, or biological material

Policy information about studies with human participants or human data. See also policy information about sex, gender (identity/presentation), and sexual orientation and race, ethnicity and racism.

Reporting on sex and gender	Not applicable
Reporting on race, ethnicity, or other socially relevant groupings	Not applicable
Population characteristics	Not applicable
Recruitment	Not applicable
Ethics oversight	Not applicable

Note that full information on the approval of the study protocol must also be provided in the manuscript.

Field-specific reporting

Please select the one below that is the best fit for your research. If you are not sure, read the appropriate sections before making your selection.

☐ Life sciences ☐ Behavioural & social sciences ☒ Ecological, evolutionary & environmental sciences

For a reference copy of the document with all sections, see nature.com/documents/nr-reporting-summary-flat.pdf

Ecological, evolutionary & environmental sciences study design

All studies must disclose on these points even when the disclosure is negative.

Study description	This study was observational and descriptive in nature, based on environmental sampling from a single subglacial Antarctic lake (Subglacial Lake Mercer). An environmentally clean hot water drilling system was used to penetrate ~1.1 km of ice to sample water and sediments of the lake. Quantitative genomic data were derived from 1,374 single-cell amplified genomes obtained from one lake water cast, one sediment core, and one bulk sediment sample. No treatment factors or experimental manipulations were applied; therefore, no factorial, nested, or hierarchical design was used. The experimental units were the 1,374 individual microbial cells, and data were analyzed as single-replicate environmental samples.
Research sample	Lake water was sampled at mid-depth of the ~15 m water column and concentrated in-situ using filters with pore sizes of 3.0, 0.8, and 0.2 um. A sediment catcher was used to sample bulk surface sediment. Five sediment core samples (10 cm depth, sectioned at 2 cm intervals) were obtained from a sediment core.
Sampling strategy	The lake was directly accessed through a ~0.4 m diameter borehole melted through 1,087 m of ice with an environmentally clean, hot water drilling system. A large volume water filtration system (WTS-LV; McClane Inc.) was deployed to sample microbial cells and particulates in the lake water column. A sediment catcher constructed out of polyethylene was affixed at the base of the WTS-LV frame to facilitate the collection of bulk surface sediments during each cast. An Uwitec mulitcorer customized for borehole deployment was used to obtain surficial sediment cores from SLM. Samples were limited to single casts due to logistic constraints. All samples were collected aseptically and immediately cryopreserved at -80 degree Celsius. All data were collected by the Subglacial Antarctic Lakes Scientific Access (SALSA) team. Ice thickness was measured using phase-sensitive radar, and vertical profiles of conductivity, temperature, and pressure were obtained using a Seabird 19plus V2 SeaCAT profiler.
Data collection	Single-cell sorting using FACS was followed by whole genome amplification and was carried out at the Single Cell Genomic Center at Bigelow Laboratory for Ocean Science. Sequencing was conducted using the HiSeq X ten system (Illumina).
Timing and spatial scale	Mercer Subglacial Lake is located near the confluence of Mercer Ice Stream and Whillans Ice Stream. This lake has a surface area of

	ca. 143 square kilometre, and a water column depth at the lowest hydropotential was ~15 m deep. The borehole retained for 8 days since 26 December 2018. Sampling activities were conducted from 28 December 2018 to 5 January 2019, which yielded for this study: (1) a McLane WTS-LV in situ filtration cast for sampling lake water and bulk surface sediments on 30 December 2018 and (2) a UWITEC Multicorer cast for sampling sediments on 31 December 2018.
Data exclusions	Single cells that failed to provide sufficient data for successful assembly or taxonomic classification were excluded in this study.
Reproducibility	Due to the rarity of the samples and the high sequencing cost, our single-cell amplified genomes were obtained from a single water sample cast, sediment core, and bulk sediment sample. Although we were not able to compare microbial genomes between different water sampling casts and sediment cores, the genomic data analysis was accomplished in triplicate, ensuring the accuracy of our results.
Randomization	This study is limited to a single location and borehole for a subglacial Antarctic lake.
Blinding	Our single-cell genomic data were derived from a single water cast, sediment core, and bulk sediment sampling collected from a single location. Therefore, blinding was not relevant to our study.

Did the study involve field work? ☒ Yes ☐ No

Field work, collection and transport

Field conditions	Equipment (drill, science laboratories, operations structures) and fuel that was staged along the South Pole overland traverse route during the 2017-18 season was traversed the final ~150 km to SLM during the 2018-19 drilling season. A ski way was groomed that allowed LC130 and smaller ski equipped aircraft to transport scientists and operations staff (~50 total personnel) to the site. Once camp and drill set-up were complete, drilling of the main borehole began (23 December 2018) and was completed on 26 December 2018, 1087 m beneath the ice surface. This produced a ~0.4 m diameter borehole that was visually verified by a downhole camera. After eight days of subglacial water and sediment sampling, all samples, equipment, structures, and personnel on site were retrograded to the United States via McMurdo Station (Ross Island).
Location	84.640287° S, 149.501340° W (borehole location)
Access & import/export	The research in this study did not occur in an Antarctic Specially Protected Area (ASPA) and/or engage in harmful interference or collect any part of native mammals or birds, and therefore, an Antarctic Conservation Act (ACA) permit was not required. Permission to trans-ship samples from Antarctica through New Zealand to the United States was granted by a Ministry for Primary Industries permit (permit no. 2018068204) extended to Brent Christner by the government of New Zealand.
Disturbance	Action-specific measures were designed, tested, and implemented to prevent contamination of subglacial microbial communities by non-native species or other foreign substances. This was accomplished using environmentally clean drilling technology and by disinfecting all equipment before its deployment in the borehole. Although some effects of the activities may be detectable for a long period of time after drilling (e.g. greywater disposal near camp facilities), the overall impacts were localized in proximity to the drill site and did not result in a measurable adverse impact on the Mercer Ice Stream or adjacent Ross Ice Shelf. The activities associated with drilling and operation of the camp facilities were within the typical range of USAP activities conducted each year and did not significantly increase impacts already being realized because of USAP field activities. An environmental risk assessment by the NSF concluded that our research activities had a minor or transitory effect on the West Antarctic surface and subsurface environments we disturbed during the period of research (December 2018 to January 2019).

Reporting for specific materials, systems and methods

We require information from authors about some types of materials, experimental systems and methods used in many studies. Here, indicate whether each material, system or method listed is relevant to your study. If you are not sure if a list item applies to your research, read the appropriate section before selecting a response.

Materials & experimental systems

n/a	Involved in the study	
☒	☐	Antibodies
☒	☐	Eukaryotic cell lines
☒	☐	Palaeontology and archaeology
☒	☐	Animals and other organisms
☒	☐	Clinical data
☒	☐	Dual use research of concern
☒	☐	Plants

Methods

n/a	Involved in the study	
☒	☐	ChIP-seq
☐	☒	Flow cytometry
☒	☐	MRI-based neuroimaging

Plants

Seed stocks	*Report on the source of all seed stocks or other plant material used. If applicable, state the seed stock centre and catalogue number. If plant specimens were collected from the field, describe the collection location, date and sampling procedures.*
Novel plant genotypes	*Describe the methods by which all novel plant genotypes were produced. This includes those generated by transgenic approaches, gene editing, chemical/radiation-based mutagenesis and hybridization. For transgenic lines, describe the transformation method, the number of independent lines analyzed and the generation upon which experiments were performed. For gene-edited lines, describe the editor used, the endogenous sequence targeted for editing, the targeting guide RNA sequence (if applicable) and how the editor was applied.*
Authentication	*Describe any authentication procedures for each seed stock used or novel genotype generated. Describe any experiments used to assess the effect of a mutation and, where applicable, how potential secondary effects (e.g. second site T-DNA insertions, mosiacism, off-target gene editing) were examined.*

Flow Cytometry

Plots

Confirm that:

- [x] The axis labels state the marker and fluorochrome used (e.g. CD4-FITC).
- [x] The axis scales are clearly visible. Include numbers along axes only for bottom left plot of group (a 'group' is an analysis of identical markers).
- [ ] All plots are contour plots with outliers or pseudocolor plots.
- [ ] A numerical value for number of cells or percentage (with statistics) is provided.

Methodology

Sample preparation	Lake water samples (1 mL each) were supplemented with 5% (v/v) glycerol and 1× Tris-EDTA buffer (final concentrations) and stored at −80 °C until further analysis. For sediment samples, approximately 5 g was mixed with 20 mL of sterile-filtered phosphate-buffered saline (PBS), vortexed for 30 seconds, and centrifuged at 2,500 × g for 30 seconds to remove large particles. The resulting supernatant was then supplemented with 5% (v/v) glycerol and 1× TE buffer, and stored at −80 °C under the same conditions as the lake water samples.
Instrument	BD Influx Mariner flow cytometer
Software	BD Sortware software
Cell population abundance	*Describe the abundance of the relevant cell populations within post-sort fractions, providing details on the purity of the samples and how it was determined.*
Gating strategy	Cells were initially gated (P1 gate) based on cell size (FSC) and nucleic acid content (531 nm fluorescence intensity). Subsequently, populations from the P1 gate were further refined (P2 gate) by selecting cells with relatively high SYTO 9 fluorescence at 531 nm and low autofluorescence at 692 nm.

- [x] Tick this box to confirm that a figure exemplifying the gating strategy is provided in the Supplementary Information.
